# Single‐cell transcriptome atlas of human mesenchymal stem cells exploring cellular heterogeneity

**DOI:** 10.1002/ctm2.650

**Published:** 2021-12-29

**Authors:** Zheng Wang, Chengyan Chai, Rui Wang, Yimei Feng, Lei Huang, Yiming Zhang, Xia Xiao, Shijie Yang, Yunfang Zhang, Xi Zhang

**Affiliations:** ^1^ Medical Center of Hematology the Second Affiliated Hospital Army Medical University Chongqing China; ^2^ State Key Laboratory of Trauma Burn and Combined Injury Army Medical University Chongqing China; ^3^ Department of Urology the Second Affiliated Hospital Army Military Medical University Chongqing China; ^4^ Department of Plastic and Cosmetic Surgery the Second Affiliated Hospital Army Medical University Chongqing China; ^5^ Time Plastic Surgery Hospital Chongqing China; ^6^ National Clinical Research Center for Hematologic Diseases the First Affiliated Hospital of Soochow University Suzhou China

**Keywords:** extracellular matrix, heterogeneity, mesenchymal stem cells, single‐cell RNA sequencing

## Abstract

**Background:**

The heterogeneity of mesenchymal stem cells (MSCs) is poorly understood, thus limiting clinical application and basic research reproducibility. Advanced single‐cell RNA sequencing (scRNA‐seq) is a robust tool used to analyse for dissecting cellular heterogeneity. However, the comprehensive single‐cell atlas for human MSCs has not been achieved.

**Methods:**

This study used massive parallel multiplexing scRNA‐seq to construct an atlas of > 130 000 single‐MSC transcriptomes across multiple tissues and donors to assess their heterogeneity. The most widely clinically utilised tissue resources for MSCs were collected, including normal bone marrow (*n* = 3), adipose (*n* = 3), umbilical cord (*n* = 2), and dermis (*n* = 3).

**Results:**

Seven tissue‐specific and five conserved MSC subpopulations with distinct gene‐expression signatures were identified from multiple tissue origins based on the high‐quality data, which has not been achieved previously. This study showed that extracellular matrix (ECM) highly contributes to MSC heterogeneity. Notably, tissue‐specific MSC subpopulations were substantially heterogeneous on ECM‐associated immune regulation, antigen processing/presentation, and senescence, thus promoting inter‐donor and intra‐tissue heterogeneity. The variable dynamics of ECM‐associated genes had discrete trajectory patterns across multiple tissues. Additionally, the conserved and tissue‐specific transcriptomic‐regulons and protein‐protein interactions were identified, potentially representing common or tissue‐specific MSC functional roles. Furthermore, the umbilical‐cord‐specific subpopulation possessed advantages in immunosuppressive properties.

**Conclusion:**

In summary, this work provides timely and great insights into MSC heterogeneity at multiple levels. This MSC atlas taxonomy also provides a comprehensive understanding of cellular heterogeneity, thus revealing the potential improvements in MSC‐based therapeutic efficacy.

## Graphical abstract

Graphical Abstract.

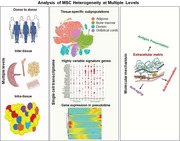

## 1 INTRODUCTION

Tissue engineering and regenerative medicines utilising mesenchymal stem cells (MSCs) are considered promising for clinical applications in various diseases.^1^ However, substantial batch‐to‐batch variations in phenotypes and functions have limited consensus, thus hampering the efficiency and reproducibility in basic research and clinical application.^2^ MSC heterogeneity at multiple levels, including variations from donors, tissues, subpopulations and individual cells, is poorly understood. Therefore, it is important to understand these effects on MSC‐based therapeutic efficacy systematically.

Bulk population sequencing cannot accurately reveal the information of individual cells in heterogeneous cells or tissues.^3^ Furthermore, flow cytometry using a few cell surface markers cannot fully explore cellular heterogeneity and subpopulations of MSCs. It is difficult to conduct an integrative analysis of samples from different donors or tissue sources due to assay imprecision and inconsistency. Furthermore, proliferative competition among different subpopulations can change their proportions, eliminating disadvantaged subpopulations.^4^ However, a comprehensive transcriptomic landscape of heterogeneous populations can analyse the alterations at single‐cell resolution due to the advances in single‐cell RNA sequencing (scRNA‐seq).^5^ Several recent scRNA‐seq studies have characterised MSC heterogeneity in a single or two tissues.^6^, ^7^, ^8^ However, donor, tissue source, culture environment, isolation methods, and passage can affect the phenotype and clinical utility of MSC‐base products.^2^ The comprehensive transcriptome atlas of MSCs to decipher MSC heterogeneity under a uniform system, including donor and tissue variations, has not been identified due to the limited scales or variable techniques in MSC isolation, culture and storage in different laboratories.

Extracellular matrix (ECM) of MSCs generate well‐defined scaffolds in tissue engineering via its structural and signalling functions.^9^ Besides constructing tissue architecture by providing highly organised macromolecules, recent studies have shown that ECM in MSC regulates their potential roles in immunomodulation,^10^ oxidative resistance^11^ and ageing.^12^ Furthermore, ECM also determinates MSC lineage differentiation via multiple mechanisms.^13^ However, there remains a lack of knowledge on the functional contribution of ECM to cellular heterogeneity.

MSCs from different origins have heterogenous functions on immunomodulation. Besides several registered clinical trials, many countries have approved MSC transplantation to treat immune disorder diseases, such as asthma, stroke and graft‐versus‐host disease (GVHD), due to their immunosuppressive property.^14^, ^15^ MSCs, as unconventional antigen‐presenting cells, stimulate T‐cell proliferation by secreting pro‐inflammatory factors.^15^ However, no study has reported this phenomenon, especially in the underlying mechanisms controlling uniformity and heterogeneity of inter‐ and intra‐ tissue MSCs.

This study analysed more than 130 000 single MSCs from multiple tissues in 11 normal donors using a droplet‐seq approach. This study also demonstrated a global transcriptional heterogeneity across different tissues and donors. The tissue‐specific and conserved MSC subpopulations were identified and characterised. ECM predominantly determined MSC heterogeneity. Furthermore, ECM‐related inflammation, ageing, antigen‐processing‐and‐presentation were highly heterogeneous in tissue‐specific subpopulations. The construction of developmental trajectories also illustrated inter‐ and intra‐ tissue heterogeneity of MSCs. This work comprehensively augments the resource value of MSC subpopulations, thus promoting novel quality control and therapeutic strategies of MSC products.

## 2 METHODS

### 2.1 Isolation and culture of human MSCs

Adipose tissues were obtained from specimens of three female healthy donors (A01, A02 and A03) who underwent surgical liposuction for cosmetic purposes. Liposuction product was minced using a surgical scalpel and digested with commercial .1% collagenase IV (Sigma, C5138) in sterilised 1x phosphate‐buffered saline (PBS) containing 1% bovine serum albumin (BSA; Sangon Biotech) while continuously shaking for 60 min at 37°C. Cells were seeded in a T25 culture flask after Ficoll density separation (Sigma, F5415). Adipose‐derived MSCs were isolated as described in the previous report.^16^

Bone marrow specimens were collected from three healthy donors (B01, B02 and B03) via aspiration. The mononuclear cells were isolated using Percoll density centrifugation (Sigma, P1644) for MSC isolation. BMSCs were separated from other hematopoietic cells through their plastic adherence in culture.

Foreskin specimens of three young male donors (D01, D02 and D03) were obtained after circumcision. The specimens were cut into 4–6 mm^2^ pieces, after removing

adipose and connective tissue using scissors and tweezers. The dermis was then minced into 1‐mm pieces and plated onto p100 dishes after epidermis removal. Leftover tissues were incubated with Dispase II 1 mg/ml (Sigma, SCM133) at 4°C overnight. The above procedure was conducted as described in the previous report.^17^ Fetal bovine serum was added dropwise on each tissue piece, then incubated at 37°C.

MSCs were isolated from the umbilical cords of two healthy pregnant women (U01 and U02) during caesarean delivery as described in the previous report.^18^ The umbilical cord tissue mechanically dissociated into about 1–2 mm^2^ pieces after dissection. The samples were then seeded into T75 culture flasks with .1% collagenase I (Sigma, C0130) at 37°C while shaking.

Antibiotics (1%) and 10% fetal bovine serum (Hyclone) with 1 ug/100 ml basic fibroblast growth factor (bFGF) (HEGFP‐0602; Cyagen) were added into MSC culture medium in a humidified incubator at standard cultivation conditions (37°C, 5% CO_2_). The medium was changed every 2–3 days. The plates were monitored every day until the cells reached confluence. The cells were passaged after reaching 80%–90% confluence.

### 2.2 Lineage differentiation in vitro

The confluent cells from the individual donors were treated with MSC osteogenic differentiation medium (Catalogue No. HUXMA‐90021; Cyagen) for 3 weeks for osteogenic induction. The medium was then removed from the wells, and the cells were fixed with 4% formaldehyde solution in PBS for 30 min. The induced cells were then stained with 1 ml of alizarin red S solution (Catalogue No. S0141; Cyagen) for 30 min. To induce adipogenesis, the cells were treated with in human MSC adipogenic differentiation medium (Catalogue No. HUXMA‐90031; Cyagen) to induce adipogenesis. The medium was removed from each well after differentiation, and then the cells were fixed with 4% formaldehyde solution for 30 min. The cells were stained with 1 ml of oil red O working solution (Catalogue No. S0131; Cyagen) for 30 min. All the procedures were followed by the manufacturer's manuals. Chondrogenic differentiation medium OriCell was prepared with ITS additives, dexamethasone, ascorbic acid, sodium pyruvate, TGFb3 and proline. MSCs (8 × 10^5^) were then re‐suspended in a 1 ml chondrogenic differentiation medium on the first day. MSC suspension (.5 ml) was added to each 15 ml polypropylene tube, then centrifuged at 300 x g for 5–10 min at room temperature. The caps of 15 ml centrifugal tubes were loosened to allow gas exchange. Cell pellets were incubated at 37°C and 5% CO₂ for 2–3 weeks. The chondrogenic differentiation medium was replaced every 2 days until one micro ball formed (about 1 or 2 mm in diameter). The micro balls were then fixed with 4% PFA and stained with Alcian Blue, according to manual instructions.

### 2.3 Flow cytometry sorting and analysis

The MSCs for individual donors were trypsinised with TrypLE Express (Thermo Fisher Scientific, USA) and washed twice with PBS at the first or second passage. Flow cytometry was then used to sort the MSCs to detect specific surface markers (positive for CD90, CD73 and CD105, and negative for CD11b, CD19, CD34, CD45 and HLA‐DR), according to instructions (BD Stemflow, 562245). At least 10 000 events were obtained on a FACSVerse instrument (BD Bioscience), and the results were analysed using FlowJo software (Tree Star, Ashland, OR).

### 2.4 scRNA‐seq library preparation and sequencing

The high‐quality cultured MSCs were used for scRNA‐seq. All the samples used for single‐cell transcriptome underwent quality control to confirm their tri‐lineage differentiation ability and the identity of surface markers. MSCs were digested and re‐suspended in 1x PBS with .04% BSA after centrifuging at 300 g for 5 min. The cells were filtered using a 40‐μm strainer and purified via flow cytometry sorting. Corning Cell Counter was used to determine the cell concentration. Flow cytometry sorting removed a few dead cells (less than 5%) after 4′,6‐diamidino‐2‐phenylindole staining. MSCs with high (above 95%) cell viability were stained with trypan blue staining, then washed twice and re‐suspended using cold 1x PBS after sorting to reduce background noise caused by cell‐free RNA. The number of cells loaded into each channel was strictly controlled (about 10 000—11 000 cells) to minimise doublets. Briefly, cells were examined under a microscope, then loaded in each channel with a target output of 3,000 to 6000 cells. According to the 10x official statement on doublet rate, it was less than 5% (roughly from 3.9% to 4.6%). The 10x Genomics Chromium platform was used to capture the single barcoding cells in order and generate the single‐cell gel beads‐in‐emulsion (GEMs). scRNA‐seq libraries were constructed using 10x Genomics Chromium Single Cell 30 v2 Reagent Kit, single‐cell 3′ Chip Kit v2 (PN‐120236) and i7 Multiplex Kit (PN‐120262), following the manufacturer's protocol. Each sample occupying at least two channels and outputs were merged after GEMs generation. Final library quality was evaluated on the Agilent Bioanalyzer using a High Sensitivity DNA Kit (Agilent Technologies, Santa Clara, CA, USA) after reverse transcription and library preparation on C1000 Touch Thermal cycler with 96‐Deep Well Reaction Module (Bio‐Rad). The libraries were sequenced on an Illumina NovaSeq 6000 System in a 2 × 150 bp paired‐end mode (Oebiotech, Shanghai).

### 2.5 Single‐cell demultiplexing, barcode processing and unique molecular identifier counting

The official 10x Genomics pipeline Cell Ranger v2.1.0 (https://support. 10xgenomics.com/single‐cell‐geneexpression/software/pipelines/latest/what‐is‐cell‐ranger) was used for cell demultiplexing, aligning to the human genome, version GRCh38, barcode processing and unique molecular identifier (UMI) counting. STAR aligner was used to map reads on the human genome and transcriptome.^19^ The generation of the gene‐barcode matrix only depends on the accurately mapped, non‐polymerase chain reaction (PCR) duplicates with valid barcodes and UMIs. The distribution of detected genes per cell was calculated to exclude multiple cells or doublets, or extreme outliers in terms of library complexity. The cells with less than 1000 and more than 5000 detected genes were excluded (representing 2%–6% of total detected cells). The cells with less than 500 UMI count or more than 10% of the transcripts from mitochondrial genes were also removed. Quality control (QC) also filtered out genes detected in less than five cells. Cells with UMI/gene numbers out of the limit of mean value +/– 2‐fold of standard deviations based on a Gaussian distribution of each cells' UMI/gene numbers were also removed. The resulting gene‐cell UMI count matrices for each sample were merged into one matrix using the cellranger aggr pipeline. This pipeline also normalised the libraries to the same sequencing depth, producing a matrix of gene counts versus cells. Library size normalisation on the filtered matrix was performed in Seurat v2.3.0 to obtain a normalised count.^20^

### 2.6 Dimensionality reduction and clustering

Top variable genes across single cells were identified using the earlier described method.^21^ Briefly, the average expression and dispersion of each gene were calculated. Genes were subsequently placed into several bins based on the expression. Principal component analysis (PCA) was used to reduce the dimensionality on the log‐transformed gene‐barcode matrices of top variable genes. The suitable number of dimensions was analysed by ElbowPlot and JackStrawPlot in Seurat first.^20^ A suitable principal component (PCs) with statistical significance was selected based on Elbowplot and JackStrawPlot. PC25 was selected for downstream analysis. Highly variable genes were identified and used as input to the mnnCorrect function for batch correction. FastMNN from MNN Correct algorithm^22^ was then performed on the PC output, where nearest neighbours were determined in the PCA dimensionally‐reduced space. After multi‐sample PCA dimension reduction, the multiBatchPCA function from the Scran package (version 1.4.5)^23^ was then used to improve neighbour detection. Visualisation and evaluations of batch‐corrected output were conducted using a tSNE plot. The cells were clustered based on a graph‐based clustering approach and were visualised in 2D using tSNE. Likelihood ratio test that simultaneously calculated the changes in mean expression and percentage of expressed cells was used to identify significantly differentially expressed genes among clusters, using the function FindAllMarkers in Seurat. A cutoff of *p*‐adj < .01 and *p* < .01 was used to filter genes. Genes expressed in a minimum of 25% of cells in either of the test populations were used for further analysis. Cyclone model was implemented in the Scran package to further confirm the cell‐cycle phases of the cells. The expression levels of a set of cell‐cycle related genes were used as input to classify cells into G1, S and G2/M phases. The model was run using the preselected human genes in the Scran package on the raw UMI matrix. DoubletFinder (R version 2.0)^24^ was used to predict doublet artefacts in the scRNA‐seq data.

### 2.7 MSC correlation score

The raw expression data obtained cell after filtering was used to compare with classic MSC from Blueprint reference by Spearman test via SingleR package.^25^ The Spearman test score was used to perform further tSNE visualisation.

### 2.8 Reconstruction of cell development trajectories

The algorithms implemented in the Monocle2 (version 2.4.0)^26^ were performed following software instructions to assess the developmental progression of MSCs across multiple subsets and order them in pseudotime. Genes used for the cell ordering were determined in an unsupervised manner based on their dispersion across cells. The likelihood ratio test was used to identify genes that differed among the clusters in a generalised linear model. The selected genes underwent dimensionality reduction and trajectory construction was performed on with default methods and parameters, using the nonlinear reconstruction DDRTree algorithm^27^ implemented in Monocle2 via reduceDimension method. The cells were ordered along the trajectory via the orderCells method with the default parameter.

### 2.9 RNA velocity analysis

The bam files were generated from the official 10x Genomics pipeline Cell Ranger v2.1.0 as input files for RNA velocity. The pipeline Velocyto.R^28^ was used for all analysis, following the software manual in this paper. The annotation process considered only reads that could be uniquely mapped. Reads with multiple mappings or mapping inside repeat‐masked regions were removed with University of California Santa Cruz (UCSC) genome browser repeat masker output as reference.

### 2.10 Pathway and functional enrichment analysis

Go analysis and Canonical Pathways analysis of the highly differentially signature genes (log *FC* > .25, *p* < .01 and *p*‐adj < .01) in each cluster was conducted using Metascape.^29^ Go and Canonical Pathway analysis’ results were visualised using Cluster 3.0^30^ and Java TreeView (version 1.6). Metascape was also used to represent terms in a functional enrichment network with the best *p*‐values of 20 clusters with default parameters. The networks were modified and visualised using Cytoscape^31^ (version 3.7.1). TRRUST, a transcription factor‐target interaction database based on text mining and manual curation was used for regulon analysis.^32^ Pheatmap (version 1.0.12) package was used to visualise the heatmap showing data from TRRUST output. Protein‐protein interaction networks were determined using BioGrid,^33^ InWeb_IM^23^ and OmniPath^34^ databases from metascape with default parameters. Venny 2.1.0 software (https://bioinfogp.cnb.csic.es/tools/venny/) was used to draw the Venn diagram.

### 2.11 Western blot

Trans‐Blot Transfer system (Bio‐Rad Laboratories) was used to transfer proteins to PVDF membranes. The PVDF membranes were then blocked with soluble 5% dry milk in 1x TBST (Tris‐buffered saline with 0.1% Tween‐20) for 1–2 h at room temperature and then incubated with primary antibody at 4°C overnight. The antibodies included TNFa, MMP‐3, MCP‐1(1:1000 dilution, Cell signalling technology), a‐tubulin (1:1000 dilution, Proteintech), and peroxidase‐conjugated secondary antibodies (1:1000 dilution, Proteintech). The PVDF membranes were then incubated with secondary antibodies. Images were obtained with image lab software using ChemiDoc charge‐coupled device (CCD) camera (Bio‐Rad Laboratories).

### 2.12 Senescence assay

MSCs cultured in 6‐Well‐Cell‐Culture‐Plates (Corning) from each donor were passaged at P6. Senescence β‐galactosidase (SA‐β‐gal) staining was then performed using SA‐β‐gal Assay Kit (C0602, Byeotime Biotechnology), following the manufacturer's protocol. Briefly, MSCs were fixed with 4% PFA and then stained with SA‐β‐gal. Three donor‐derived MSCs were collected for each tissue for biological replicates. Seven random x200‐optical fields were acquired per donor for staining analysis. The average percentage of SA‐β‐gal‐positive MSCs for each donor was used for group statistical analysis.

### 2.13 Bulk RNA‐seq library preparation and data analysis

TRIzol reagent (Invitrogen, USA) was used to extract total RNA, following the manufacturer's protocol. NanoDrop 2000 spectrophotometer (Thermo Fisher Scientific) and Qubit 2.0 (Thermo Fisher Scientific) were used to assess RNA purity and quantification. RNA integrity was assessed using the Agilent 2100 Bioanalyzer (Agilent Technologies). Subsequent library preparation was conducted with RNA Integrity Number (RIN) above 7. TruSeq Stranded mRNA LT Sample Prep Kit (Illumina, San Diego, CA, USA) was used to construct the libraries, following the manufacturer's instructions. The pair‐end 150 sequencing reads were generated on Illumina Hiseq x 10 platform by OE Biotech Co. Ltd (Shanghai, China). Low‐quality reads and adaptors were removed using Trimmomatic.^35^ The clean reads were mapped to the human reference genome (UCSC genome browser, hg38) using HISAT2.^36^ HTSeq‐count was performed for reading counts of each gene, using the annotated gene reference (hg38). FPKM of each gene was calculated using Cufflinks.^37^ Fragment bias was corrected to improve expression estimation.^38^ R package ggplot2 (3.0) was used for data visualisation.

### 2.14 Real‐time PCR

RNA isolation was performed as previously described.^39^ Reverse transcription was achieved using Invitrogen SuperScript III Kit (Thermo Fisher Scientific). Real‐time PCR was conducted using reagents from the SYBR green real‐time PCR kit (Thermo Fisher Scientific) and detected using StepOne Real‐time PCR System (Applied BioSystems). GraphPad Prism version 8.0 was used for quantitative analysis. All data were reported as means ± SD. The Cm values of samples were normalised to the corresponding Cm values of B‐actin. The following primers were used: OGN Forward (F), TGCCTTGATAGGAGGAAAACA; OGN Reverse (R), GATCCCCAAAAGCATTTAAGG; RUNX2 Forward (F), GGCCCTCCCTGAACTCTGCAC; RUNX2 Reverse (R), GCGGGGTGGTAGAGTGGATGGA; C/EBPB Forward (F), AACTCTCTGCTTCTCCCTCTG; C/EBPB Reverse (R), AAGCCCGTAGGAACATCTTT; Ap2 Forward (F), GGGCCAGGAATTTGACGAAG; Ap2 Reverse (R), CGCATTCCACCACCAGTTTATC; PPARG Forward (F), GTGGCCATCCGCATCTTTCAG; PPARG Reverse (R), GAAGCCTTGGCCCTCGGATATG; PDL1 Forward (F), TGGCATTTGCTGAACGCATTT; PDL1 Reverse (R), TGCAGCCAGGTCTAATTGTTTT; CTLA4 Forward (F), TGCAGCAGTTAGTTCGGGGTTGTT; CTLA4 Reverse (R), CTGGCTCTGTTGGGGGCATTTTC; IDO Forward (F), TCTCATTTCGTGATGGAGACTGC;IDO Reverse (R), GTGTCCCGTTCTTGCATTTGC; OSX Forward (F), TTCTGCGGCAAGAGGTTCACTC; OSX Reverse (R), GTGTT TGCTCAGGTGGTCGCTT; OCN Forward (F), GCTGTAAGGACATCGCCTACCA; OCN Reverse (R), CCTGGCTTTCTCGTCACTCTCA; ALP Forward (F), GCTGTAAGGACATCGCCTACCA; ALP Reverse (R), CCTGGCTTTCTCGTCACTCTCA; MMP1 Forward (F), ATGAAGCAGCCCAGATGTGGAG;

MMP1 Reverse (R), TGGTCCACATCTGCTCTTGGCA; MFAP2 Forward (F), GTCCAACAGGAAGTCATCCCAG; MFAP2 Reverse (R), CCTGTGTATGGAGTAGAGGCGG.

### 2.15 In vitro transfection with small interfering RNAs

The MSCs were transfected with small interfering RNA (siRNA) duplexes using the Lipofectamine RNAiMAX (Thermo Fisher Scientific), according to the manufacturer's protocol. The efficiency of knockdown in MSCs was measured by real‐time PCR in Supplementary Figure S8A,B. The sense strands of the targeting sequencing were GCCAGTACGCTCACTACTTTG (siRNA1), and TGCGGAAAGTGCGACTCATAC (siRNA2) for MMP1; CGTCCAGTACACCCACTATAG (siRNA1), TCGTACAGTGTGTGCCCATGA (siRNA2) for MFAP2. Cells were harvested about 48 or 72 h after transfection for the detection of real‐time PCR and Western blotting. All siRNA transfections were performed in triplicate.

## 3 RESULTS

### 3.1 Identification of tissue‐specific MSC subpopulations and gene expression signatures using single‐cell transcriptome profiling

**FIGURE 1:**
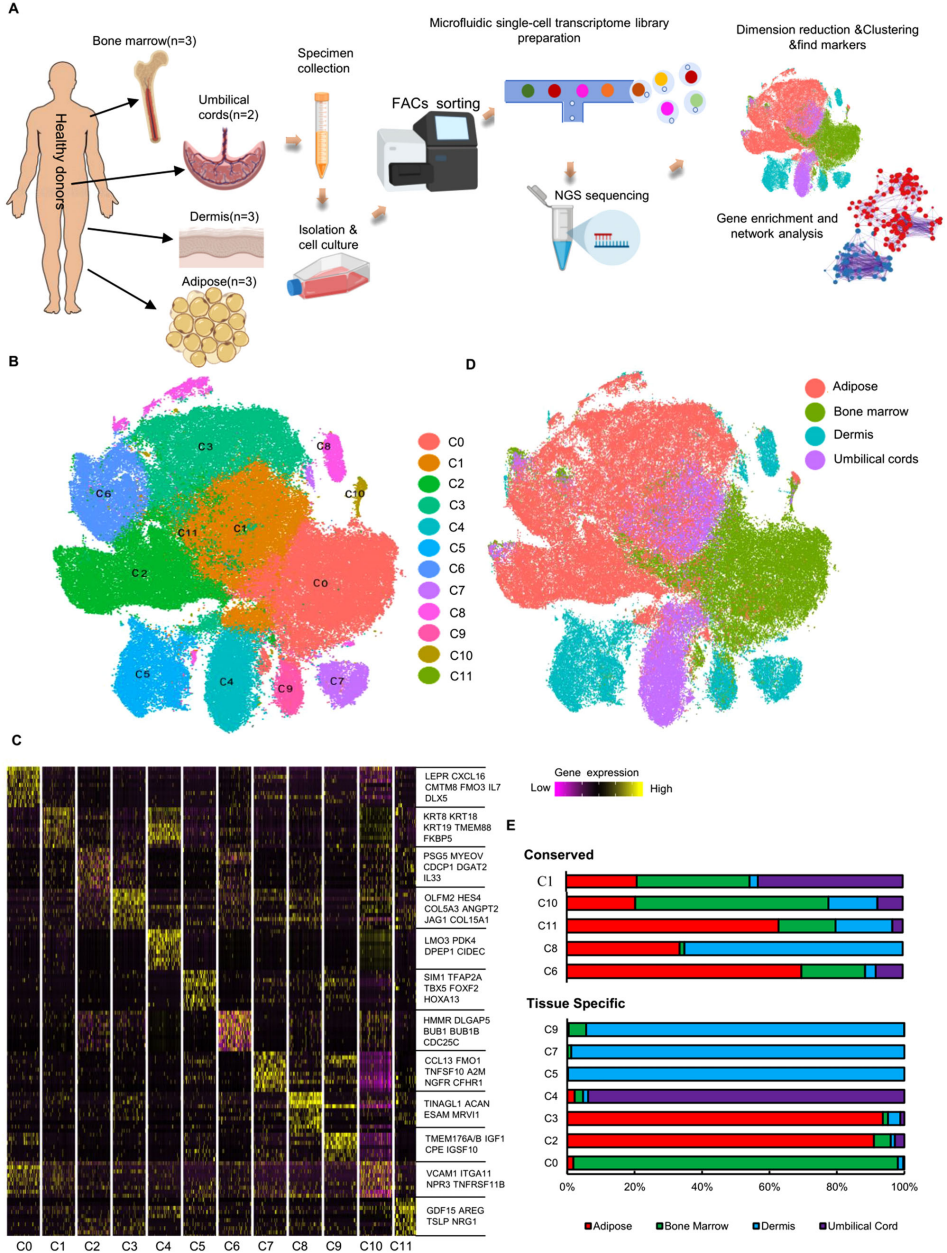
The single‐cell RNA sequencing (scRNA‐seq) analysis on tissue‐specific heterogeneity of mesenchymal stem cells (MSCs). (A) Scheme of study design. (B) 2D tSNE plot depicting 130 942 single cells, each classified into one of the 12 clusters shown with distinct colours. The number of cells for each cluster from C0 to C11 in order is 26716, 23057, 20883, 19607, 10203, 9433, 9322, 3830, 3695, 2971, 1160 and 65. (C) Gene‐expression heatmap of the top 10 marker genes for each tissue, displaying 100 randomly selected cells per cluster excluding cluster 11 (C11). Colour scale: yellow, high expression. black, low expression. (D) t‐SNE plot of multiple‐tissue derived MSCs, colour‐coded for tissue type. Adipose (*n* = 50,983), Bone marrow(*n* = 37,732), Dermis(*n* = 20,995), Umbilical cords(*n* = 21,232). (E) The relative contribution of each cluster was weighed by the number of cells per tissue and scaled to 100%.

Multiple‐lineage differentiation via in‐vitro induced conditions and specific surface markers via flow cytometry were used to validate the isolation methods and culture conditions for MSCs (Figure S1). FGF was added in the culture medium to maintain the undifferentiated proliferation and immunosuppressive properties of MSCs.^40^ To study the diversity and developmental trajectories of human MSCs, cells at early passage (P1–2) sorted by flow cytometry were then subjected to scRNA‐seq following 10x Genomics transcriptomic protocol to assess the diversity and developmental trajectories of human MSCs. An atlas with more than 130,000 single‐MSC transcriptomes from 11 normal donors (ages 22–46) and multiple tissues (adipose, bone marrow, dermis and umbilical cords) was constructed after stringent quality control and data normalisation (Figure 1A, Table S1). The identified 12 clusters based on the expression of highly variable genes (HVGs) across the total cell population were visualised using t‐distributed stochastic neighbour embedding (t‐SNE) (Figure 1B) or uniform‐manifold‐approximation‐and‐projection (Figure S2A). The diversity of MSC subpopulations had a tissue‐type‐dependent pattern, revealing that MSCs from different tissues have prominent transcriptomic heterogeneity (Figure 1C, Figure S4A). To better describe the properties of MSC subpopulations, tissue‐specific clusters were defined (the relative abundance from one tissue >90% in this cluster), and the other clusters were defined as tissue‐conserved, representing at least two of those whose proportions are relatively abundant. Each tissue had 1‐3 tissue‐specific MSC subpopulations, including C0 specific for bone‐marrow‐derived MSCs (BMSCs), C2 or C3 specific for adipose‐derived MSCs (AMSCs), C4 specific for umbilical‐cord‐derived MSCs (UMSCs) and C5, C7 or C9 specific for dermis‐derived MSCs (DMSCs) (Figure 1D). R package SingleR^25^ was used for similarity evaluation at single‐cell resolution to assess MSC heterogeneity with classic MSC transcriptome as the reference. The results revealed that C0 MSCs were significantly different from the other clusters (Figure S2B).

**FIGURE 2:**
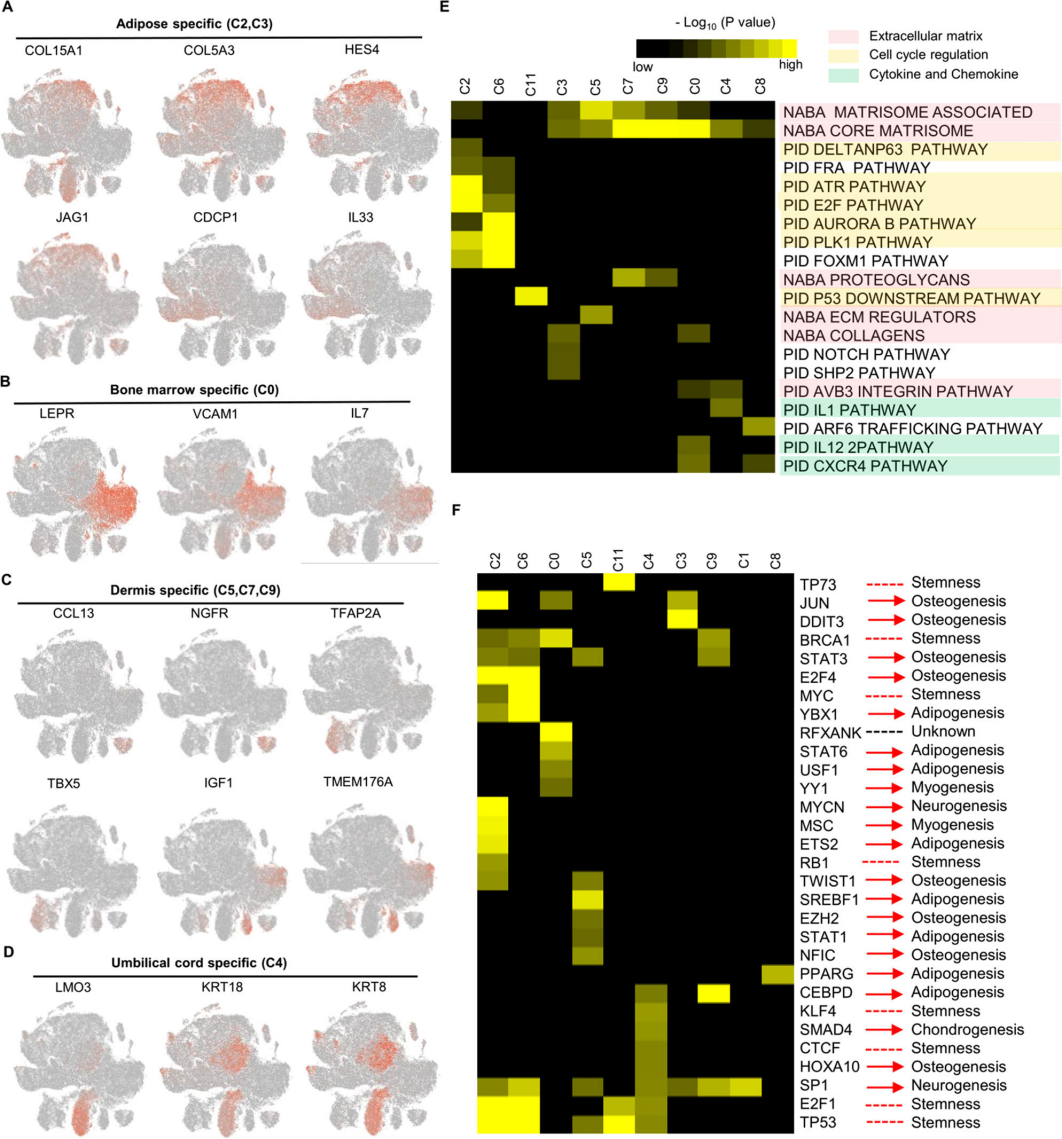
Characterization of tissue‐specific subpopulations. (A–D) t‐SNE plot of reprehensive top10 marker genes on tissue‐specific mesenchymal stem cell (MSC) clusters, respectively. (A) Adipose, (B) Bone marrow, (C) Dermis, and (D) Umbilical cord. (E) Gene Ontology analysis of highly differential expressed genes (DEGs) for each cluster. The selected statistically top 20 canonical pathways were shown and coloured by the accumulative hypergeometric *p*‐values. (F) Heatmap of the predictive regulon activity was visualised using hierarchically clustering. The potential functions related to lineage differentiation were annotated manually, according to the published references. The black to yellow colouring indicates a relative *p*‐value in the legends.

Heatmap showing the top‐10 differential expressed genes (DEGs) revealed the distinct signatures of MSCs from each cluster, via pair‐wise differential expression analysis (Figure 1E, Table S1). For instance, BMSC specific C0 subset (predominantly expressing the known MSC marker genes LEPR, CXCL12 and CXCL16) ^41^; (2) two AMSC specific subsets C2 (expressing COL15A1, COL5A3) and C3 (expressing CDCP1 and IL33); (3) UMSC specific C4 (expressing KRT8, KRT18, and LMO3); (4) three DMSC specific subsets C5 (expressing CCL13, NGFR), C7 (expressing TFP2A, TBX5), and C9 (expressing IGF1, TMEM176A/B) were identified in tissue‐specific subpopulations (Figures 1E and 2A–D). Many markers were related to ligand receptors, secreted proteins, transcriptional factors (TFs), or ECM proteins (Figure S2C–F). These genes are associated with lineage differentiation, tissue repair, and immunomodulation. For instance, LEPR,^41^ LMO3,^42^ and HES4^43^ regulate adipogenesis. TFAP2A and TBX5 also promote the maturation of Neurons^44^ and myocardial differentiation,^45^, ^46^ respectively. IGF1, highly expressed in C0 and C4, and Notch ligand Jagged1 (JAG1), highly expressed in C3, promote beneficial effects on tubular cell repair^47^ and cartilage repair,^48^ respectively. Moreover, CDCP1, IL7, CXCL16, CCL7, IL33, ITGA11, VCAM1, and TINAGL1 are involved in immune response and regulation.^49^, ^50^, ^51^, ^52^ Furthermore, doublet prediction analysis showed a sporadic distribution of doublets artefacts in our single‐cell dataset, suggesting that these clusters were not doublet or multiplet artefacts (Figure S3A).

### 3.2 ECM predominantly controls MSC heterogeneity

To better investigate the functional pathways contributing to MSC heterogeneity, Gene Ontology (GO) analysis of the predominantly significant genes was used to investigate the functional pathways promoting MSC heterogeneity. (Table S3, Figure S4B). All clusters showed different enrichment in the top 20 statistically enriched terms except for cluster (C10) with no enriched terms (all gene sets with *p*‐value >.05 threshold). The enrichment could be summarised into three main categories: (1) ECM, (2) cell cycle regulation, (3) cytokine/chemokine (Figure 2E). Terms in cell cycle regulation were only significantly enriched in C2 and C6, while those in ECM had high diversity in many subpopulations, especially in tissue‐specific subpopulations. Furthermore, cell cycle analysis indicated that the main subpopulations were in the G1 phase, implying that cell cycle variations were not the dominant factors promoting inter‐ and intra‐tissue MSC heterogeneity (Figure S3B–D).

TRRUST^32^ was used for regulon activity analysis within all TFs to decode the gene regulatory networks within each cluster. Moreover, the roles of TFs in immune regulation and lineage differentiation were manually annotated, according to previous literature. MSCs from different clusters upregulated distinct transcriptional factor regulons, including JUN,^53^ SP1^54^ and DDIT3^55^ regulons in cluster C3, and EZH2.^56^ STAT3,^57^ STAT1,^58^ TWIST1^59^ and NFIC^60^ regulons in cluster C5. This indicated the potential preference of osteogenic differentiation and inhibition of the other lineage differentiation. P53, P73, BRCA1, MYC, RB1, KLF4, CTCF and E2F1, as cell cycle regulators, also regulate differentiation, quiescence and self‐renewals of stem cells.^64^, ^65^ Furthermore, Regulon RFXANK, was specificially enriched in BMSCs, enhancing major histocompatibility complex (MHC) II‐mediated antigen processing and presentation^65^ (Figure 2F).

### 3.3 The immune‐regulatory genes associated with ECM are highly heterogeneous in tissue‐specific MSC subpopulations

**FIGURE 3:**
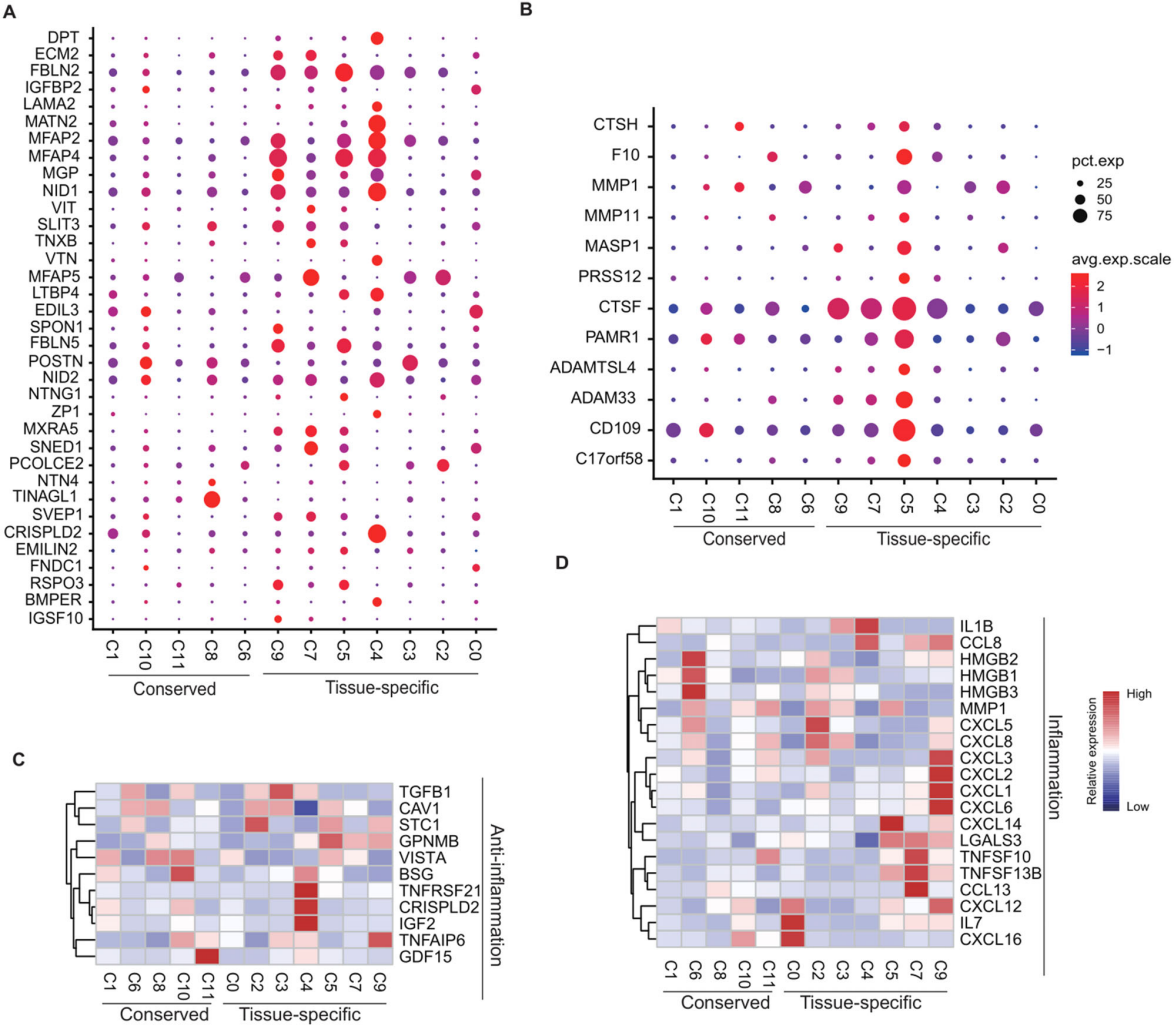
Characterisation of highly differential express genes (DEGs) in extracellur associated pathways and anti‐inflammation. Dotplot depicting the total highly DEGs in extracellur matri (ECM) glycoproteins (A) and ECM regulators (B). The size of dots encoding the percentage of cells expressing the gene. Colours correspond to the normalization of relative gene expression. Heatmap showing the differential expressed cytokines related to inflammation (C) and anti‐inflammation (D) per cluster. The average expression of genes in each cluster was row‐scaled. The colour representing the normalised average per cell gene expression level.

ECM of MSCs modulates microenvironment immune responses through immune cell migration and differentiation.^13^, ^66^ The altercation of ECM formation in MSCs can influence cytokine secretion, thus impacting on immunosuppressive properties of MSCs.^67^ The representative ECM‐associated terms were used to investigate the association between ECM heterogeneity and cytokine secretion. ECM‐associated proteins and secreted factors were significantly more heterogeneous in tissue‐specific subpopulations than that in conserved subpopulations (Figure 3A–C, Figure S4C–D). Besides chemokines, Integrins and MFAPs (microfibrillar‐associated proteins) varied in different subsets and can act as chemo‐attractants or alter immune cell behaviours.^68^, ^69^ The roles of MFAP2 and MMP1 in regulating MSC properties were also assessed. MFAP2 and MMP1 knockdown significantly increased the expression of classical immunosuppressive genes (IDO, PDL1 and CTLA4) in four types of MSCs (Figure S5). Furthermore, serials of marker genes were used to assess whether these genes can regulate adipogenesis and osteogenesis. MFAP2 and MMP1 knockdown significantly changed the expression of many genes in adipogenesis and osteogenesis. Whereas their regulation effect differed in MSCs from tissue sources based on the gene expression (Figures S6 and S7).

Besides immunosuppressive properties, MSCs can also secrete inflammatory cytokines, enhancing their immune‐stimulatory ability.^70^ The representative anti‐inflammatory and inflammatory DEGs identified by R package Seurat were also investigated (Table S4). Genes related to anti‐inflammation were highly expressed in DMSC‐ and UMSC‐ specific subpopulations. For instance, GPNMB^71^ were highly expressed in three DMSC specific subpopulations, IGF2^72^ and CRISPLD2^73^ were highly expressed in C4. However, all tissue‐specific subpopulations heterogeneously secreted higher inflammatory proteins, including (1) IL7 and CXCL16 in C0; (2) HMGB1 and HMGB3 in C2 and C3; (3) TNFSF10 and TNFSF13B in C5, C7 and C9, except for IL1B and CCL8, which were highly expressed in UMSC‐specific C4. Besides LGALS3,^74^ many inflammatory chemokines were highly expressed in DMSC‐specific subpopulations, including (1) CXCL14 in C5; (2) CCL13 in C7; (3) CXCL1, CXCL2, CXCL3 and CXCL6 in C9 (Figure 3D). Furthermore, downstream genes are heterogeneously expressed in each subpopulation in response to multiple stimulus molecules, such as cytokines (Figure S9A,B). GO analysis revealed that the terms ‘growth factors binding’ and ‘blood vessel development’ were enriched in C4, and ‘cellular responses to growth factor stimulus’ was enriched in C5 and C7 (Figure S9C,D), suggesting that their MSC homing to injured sites and response to growth factors may be more active. Next, the enriched GO terms of all DEGs with each subpopulation relating to immune response pathways were investigated. Heatmap showed that UMSC‐specific C4, conserved C1 and C8 were not significantly associated with immune response pathways, especially in immune response, compared with the other clusters (Figure S10).

### 3.4 Conserved and tissue‐specific MSC subpopulations vary in antigen processing complexes

**FIGURE 4:**
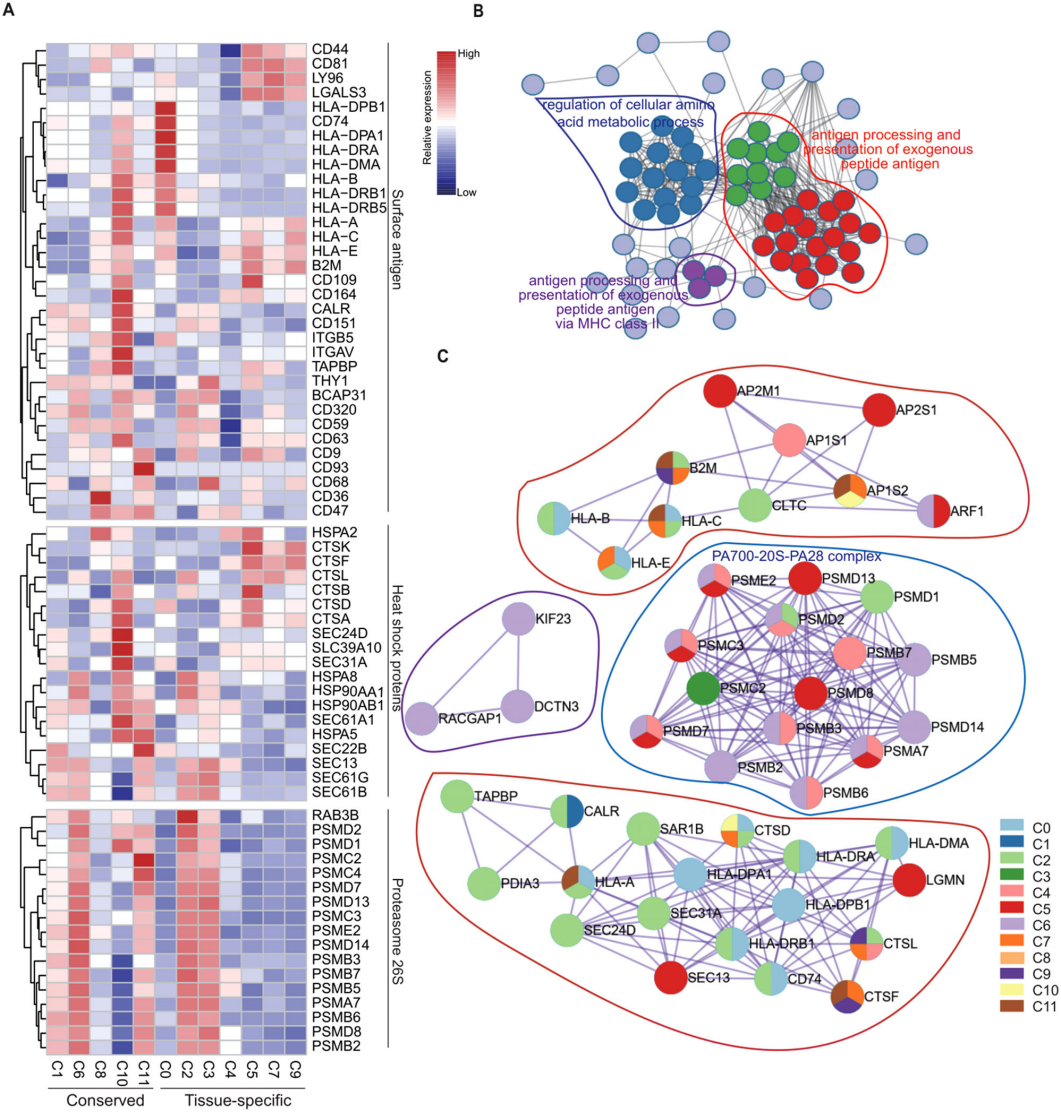
Deep transcriptome analysis exhibiting discrete expression of genes associated with antigen processing and presentation. (A) Heatmap showing the expression of highly differential express genes (DEGs) enriched by the functional blocks related to antigen processing and presentation, such as the surface antigen, heat shock proteins and proteasome 26S complexes. The colour bar representing the relative gene expression. (B) Protein‐protein interaction mapping of the selected DEGs corresponding to antigen processing and presentation. The colour representing different modules. (C) The detailed protein‐protein interaction networks were coloured by each subpopulation

The initiation of the immune response is mediated by antigen processing and presentation. Within it, ECM remodelling act as one key step.^75^ For instance, MHC class I and II relevant to antigen processing and presentation mediate inflammatory signalling transduction in ECM.^77^ To understand how ECM heterogeneity influenced the antigen processing and presentation, the expression of genes in MHC class I and II pathways were first investigated. This study showed that MHC class I and II relevant to HVGs were highly expressed in conserved and tissue‐specific subpopulations (Figure S11A,B). Many genes exhibited multifaceted protein‐protein interactions (PPIs, Figure S11C). Furthermore, within the antigen process and presentation, three functional complexes related to surface antigen, heat shock protein family, and proteasome 26S subunit exhibited heterogeneity in gene expression (Figure 4A). They were enriched in three GO terms, especially those highly associated with exogenous‐peptide‐antigen processing and presentation (Figure 4B). Functional complexes, such as PA700‐20S‐PA28 proteasome complex‐forming as an immunoproteasome, were enriched in BMSC‐specific C0, AMSC‐specific C2 and conserved C6, thus promoting the antigen process^76^ (Figure 4C). Taken together, these results suggest that UMSC‐specific C4 and conserved C8 have weak antigen‐processing and presentation ability.

### 3.5 Ageing‐related ECM proteins exhibit heterogeneous expression in tissue‐specific subpopulations

**FIGURE 5:**
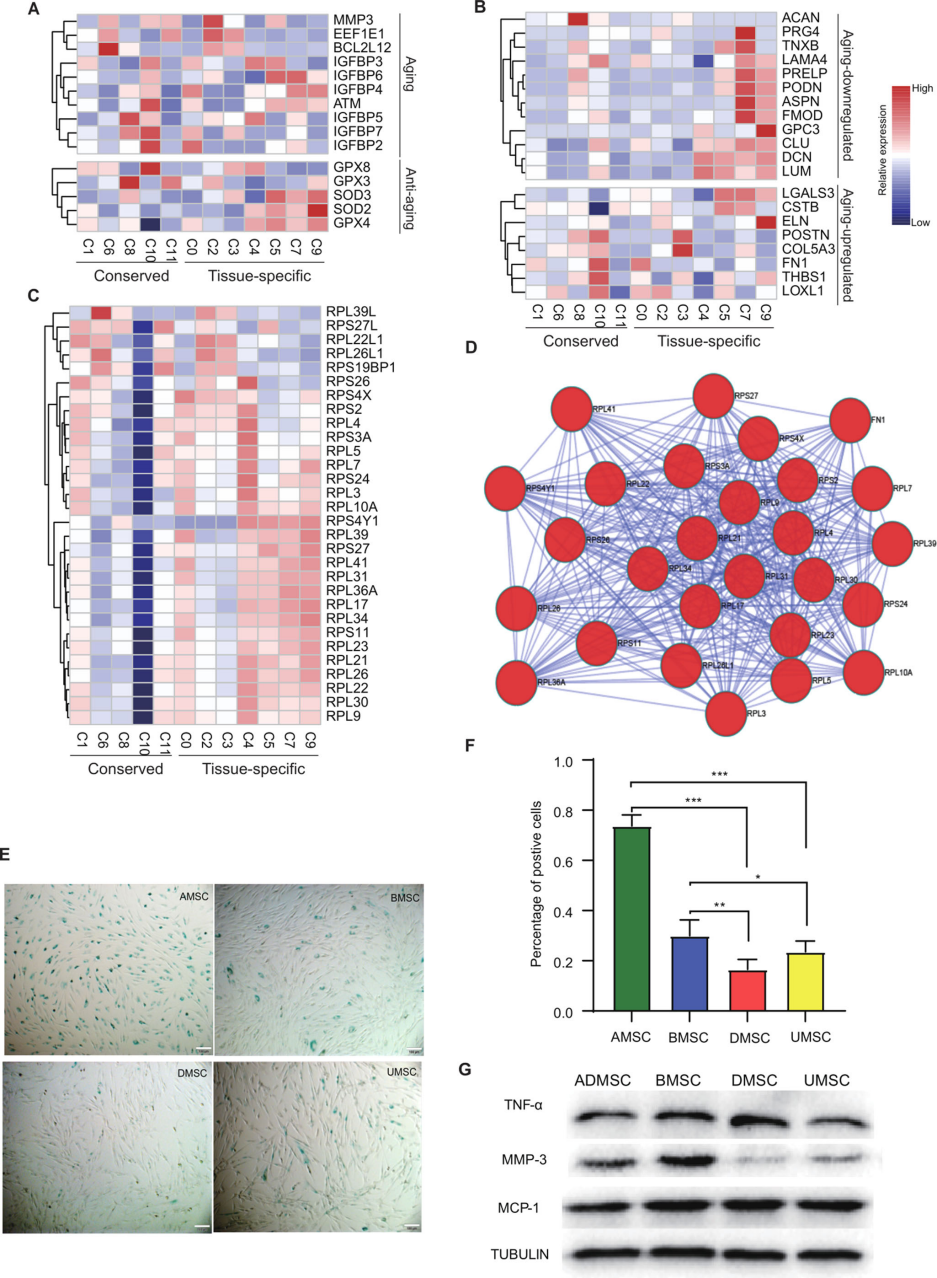
Ageing‐related heterogeneity revealed by single‐cell transcriptomes within conserved and tissue‐specific subpopulations. Using gene set enrichment analysis (GSEA) database to enrich ageing‐related genes. Heatmap showing the relative expression of ageing‐regulated genes (A), and down‐ or up‐regulated extracellular matrix (ECM) genes after ageing (B) within each subpopulation. (C) Heatmap showing the relative expression of ribosomal protein complexes from differential expressed genes (DEGs). The colour bar representing the relative gene expression. (D) Protein‐protein interaction mapping of these DEG genes. (E) Representative images of senescence‐associated‐β‐galactosidase (SA‐β‐gal) staining in multiple types of mesenchymal stem cells (MSCs). (F) Quantitative analysis of SA‐β‐gal staining. More than five fields were selected to calculate the percentage of positive cells for each donor. Each type of MSCs were collected from three donors (mean + SEM, *p  <  0.05, **p  <  0.01 and ***p  <  0.001 by two‐way ANOVA test). (G) Representative western blotting showing related expression of TNF‐a, MMP3, and MCP1, compared to a‐tubulin in different types of MSCs.

Ageing MSCs exhibiting senescence‐associated secretory phenotype (SASP) can oversecrete many pro‐inflammatory cytokines, which disturb their effect on immunosuppressive ability.^78^ The plotted heatmap showed that the expression of ageing‐related genes exhibited heterogeneously. Many anti‐ageing genes expressed higher in UMSC‐ and DMSC‐specific subpopulations as compared to the other conserved and tissue‐specific subpopulations (Figure 5A). Furthermore, the ageing‐downregulated ECM genes were highly expressed in UMSC and DMSC specific subpopulations, while the ageing‐upregulated ECM genes were expressed relatively lower in several subpopulations. In particular, UMSC specific C4 exhibited the lowest expression in ageing‐upregulated ECM genes (Figure 5B). The protection of ribosomal genes emerged as the critical prevention of cellular ageing.^79^ Methylation of ribosomal RNA genes at promoter regions increased to reduce gene expression during ageing.^80^ We found that ribosomal proteins were highly expressed in UMSC‐ and DMSC‐specific subpopulations, especially in C4 (Figure 5C). PPI analysis revealed that these ribosomal proteins exhibited high interactions (Figure 5D). Collectively, these results suggested that UMSC‐ and DMSC‐specific subpopulations (C4, C5, C6 and C7) have advantageous properties in anti‐ageing. To detect whether UMSCs and DMSCs have these advantageous properties, a senescence assay was performed, after passaging MSCs of each individual donor at P6. The percentages of SA‐β‐gal‐positive MSCs were significantly increased in AMSCs and BMSCs, compared to that in UMSCs and DMSCs (Figure 5E,F). Next, western blotting was performed to detect some ageing‐related genes. The results showed that MMP3 obviously decreased in UMSCs and DMSCs, and TNFa also obviously decreased in UMSCs (Figure 5G). In conclusion, our research suggested that MSCs isolated from dermis or umbilical cords were characteristics of anti‐ageing, compared to MSCs from bone marrow and adipose.

### 3.6 Resolving distinct donor variations in MSCs from multiple tissues

**FIGURE 6:**
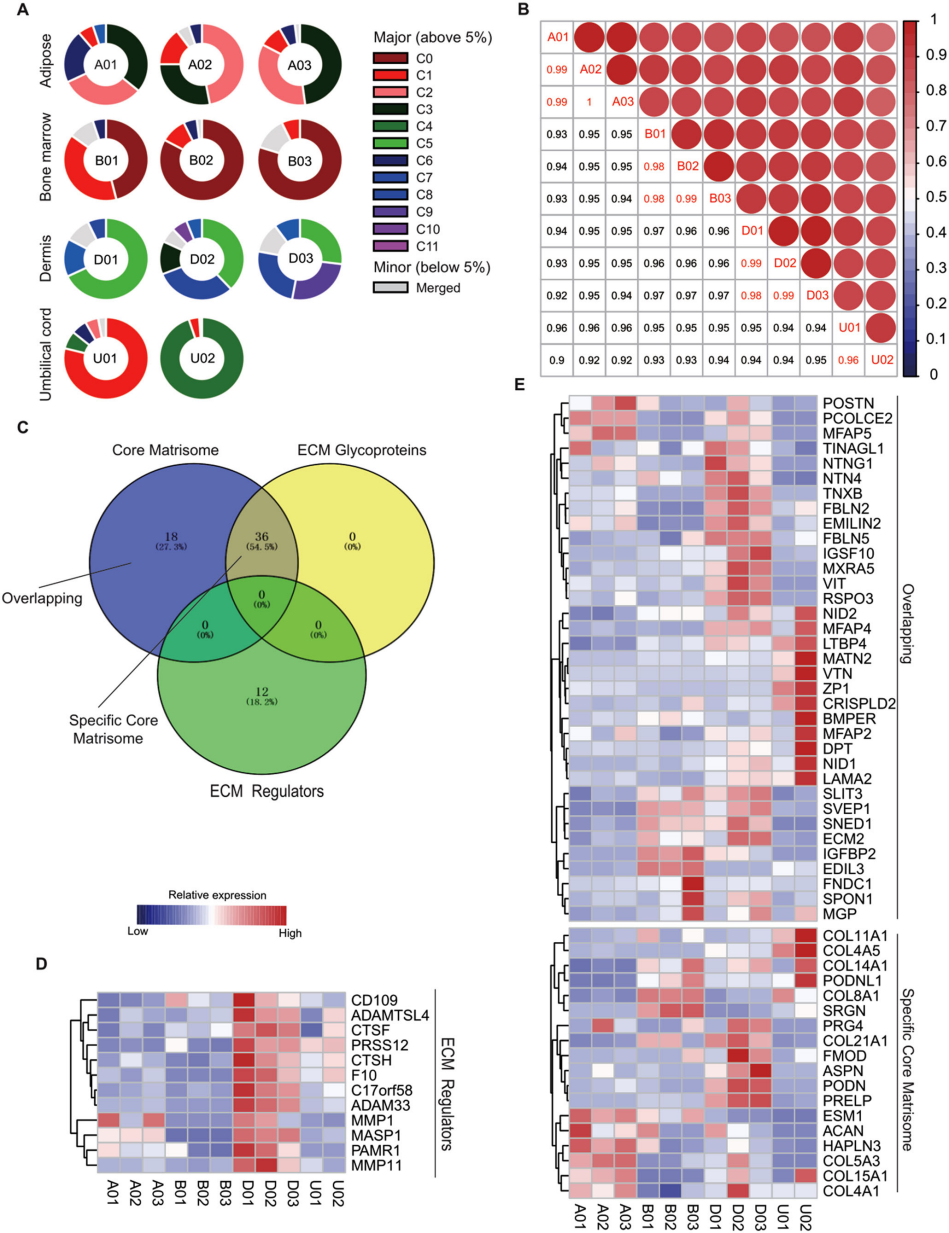
Donor‐derived heterogeneity of mesenchymal stem cells (MSCs) across multiple tissues. (A) The proportion of MSC subpopulations in each donor. The colour bar representing clusters with a high proportion (above 5%) and the grey bar encoding the merged clusters (less than 5%). (B) Correlation coefficient heatmap for different donor‐derived MSCs. The correlative value between each two‐individual donor on total differential expressed genes (DEGs). Colour legend (blue to red) encoding the value from 0 to 1. (C) Venn plot showing the overlapping DEGs in ECM associated terms. (D–E) The relative expression of genes within each ECM term: ECM regulators (D), overlapping and specific core matrisome (E). The expression was row‐scaled and coloured by low to high.

Donor variations limit the standardisation of MSC production and evaluation of MSC therapy strategies.^81^, ^82^, ^83^, ^84^, ^85^, ^86^ Herein, genes related to lineage‐differentiation and immunosuppression of MSCs were heterogeneously expressed in different tissue‐derived MSCs (Figure S8C). This study reanalysed the proportions of MSC subpopulations within individual donors, corresponding to the scRNA‐seq profiling to assess the donor‐to‐donor heterogeneity (Figure 1). The proportions in adult tissues (adipose, bone marrow and dermis) had slight donor variations, while MSCs from perinatal tissues (umbilical cords) exhibited significantly distinct heterogeneity across different donors (Figure 6A). Furthermore, the correlation coefficient between every two clusters showed high transcriptomic similarity, implying the conserved functions and evolution of MSCs. This evaluation also indicated that UMSCs from the individual donors were less correlated than the other tissue‐derived MSCs, consistent with proportion analysis (Figure 6B). Furthermore, one public UMSC dataset was downloaded and integrated to validate their heterogeneity further. There was minimal MSC mixing within three samples after batch effect correction using the MNN algorithm, similar to two UMSC samples from our dataset (Figure S8D,E). Besides UMSCs, BMSCs also exhibited distinct donor‐to‐donor heterogeneity, especially between donor B01 and two others (B02 and B03) (Figure 6A) . Furthermore, bulk RNAseq analysis indicated the relative transcriptomic stability of BMSCs at early passage (P1–P3) during passaging (Figure S8F). This result suggested that donor variations have a major effect on the phenotype and utility of MSC‐based products at an early passage, compared to cell passage.

ECM‐related pathways have dominantly controlled the heterogeneity of MSC subpopulations (Figure 2D). This study also profiled HVGs expression in three representative ECM pathways to investigate inter‐donor variations. The heatmap showed that UMSCs from individual donors were remarkably heterogeneous on ECM components, especially in ECM glycoproteins, such as LAMA2, VTN, MFAP4 and DPT (Figure 6C–E). Inter‐donor heterogeneity on the ECM‐related inflammation, antigen‐processing‐and‐presentation, and ageing was then investigated. UMSCs had a strong inter‐donor variable at these functions. However, UMSCs had anti‐ageing and immunosuppression properties based on the expression of related genes (Figure S12). Furthermore, genes in MHC I or II complex heterogeneously expressed inter‐donor in several tissues, especially in the umbilical cord (Figure S13).

The lineage‐differentiation genes of each donor were also explored. The expression of genes related to osteogenesis, chondrogenesis, and adipogenesis in individual cells had a similar distribution in AMSCs, BMSCs and DMSCs. However, multiple genes in UMSCs, such as RUNX2, CD44, CD151, and CCDC80, had substantial donor‐heterogeneity. (Figure S14A). Furthermore, all MSC cells from individual donors for each tissue were placed on their trajectories along the pseudotime to compare the stemness of different‐donor derived MSCs. The reconstruction of development trajectories in each tissue showed that UMSCs had high donor variations based on transcriptomic changes (Figure S14B). Collectively, this study that UMSCs from perinatal tissue have the highest donor variations, compared with other MSCs from adult tissues.

### 3.7 Diverse inter‐ and intra‐tissue transcriptomic regulons and PPI networks

**FIGURE 7:**
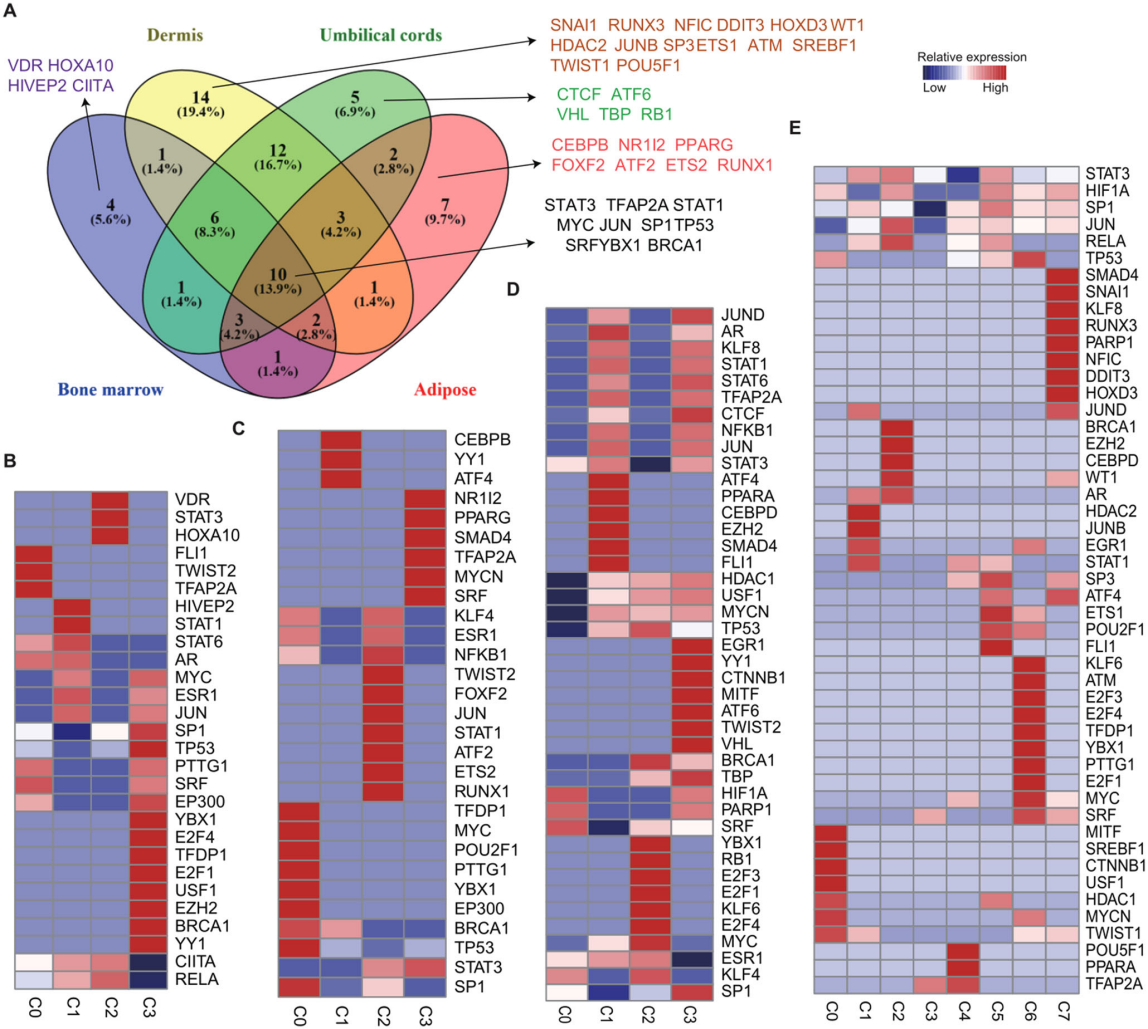
Inferred transcriptional factor regulatory networks interpreting inter‐ and intra‐tissue mesenchymal stem cell (MSC) heterogeneity. Regulons are hierarchically clustered based on the activation pattern of transcriptional factors using TRRUST. (A) Venn diagram shows the tissue‐specific and overlapping transcription factors (TFs) by Venny. (B–E) Heatmap shows the *p*‐value for detailed TFs in intra‐subsets from each tissue. (B) Adipose, (C) Bone marrow, (D) Umbilical cords and (E) Dermis. The colour scale above the heatmap showed a range of –log *p‐value*. Red, high expression; blue, low expression.

The unbiased clustering for MSCs of each tissue was performed to better investigate intra‐tissue MSC heterogeneity (Figures S15–18). Most identified top 10 marker genes for each tissue MSC subpopulation were related to ECM. Similarly, with inter‐tissue heterogeneity (Figure 2), ECM related genes also featured as specific markers to distinguish MSC intra‐tissue subpopulations, such as (1) AMSCs: HMMR, COL15A1, HLA‐DRA and CDCP1 (Figure S15B); (2) BMSCs: ITGB8, HLA‐DMB, TMEM176A and TMEM176B (Figure S16B) (3) DMSCs: ITGA7, HMMR, COL11A1, TINAGL1 and MGP (Figure S17D); (4) UMSCs: DPT, COL15A1, CLDN11 and TIMP3 (Figure S18C), were also used as specific markers to distinguish MSC intra‐tissue subpopulations. TRRUST^32^ was used for regulon activity analysis on intra‐tissue MSC subpopulations for each tissue to further explore the similarity and difference between inter‐ and intra‐tissue activity of transcriptomic regulons. Most intra‐tissue MSC subpopulations within each tissue had their specific regulons (Figure 7B–E). Venn diagram analysis was used to compare inter‐tissue differences further. Venn diagram showed the overlapping of total combined TFs from each tissue, indicating that each tissue had tissue‐specific regulons, besides MSC conserved regulons (Figure 7A). For instance, TWIST1,^87^ NFIC^60^ and DDIT3^88^ regulons were specifically enriched in DMSCs, while CEBPB,^89^ PPARG^90^ and RUNX1^91^ regulons were specifically enriched in AMSCs, which were involved in adipogenesis. The conserved regulons were associated with the regulation of immunosuppression, proliferation and stem cell renewals. For instance, STAT1, STAT3, JUN, and YBX1 were related to immunosuppression.^92^, ^93^ BRCA1, MYC, and TP53 are associated with the regulation of proliferation and stem cell self‐renewals.^94^, ^95^ These conserved features might be phenotypically distinguishable from other types of differentiated cells.

This study then dissected PPI networks on tissue‐specific subpopulations, using three combined databases (BioGRID.^33^ InWeb_IM^96^ and OmniPath^34^). Each tissue had a distinct functional network (Table S5). Notably, the top one hub gene term ‘antigen processing and presentation’ were enriched in BMSC specific C4. The top three hub gene term ‘DNA replication’, ‘chromosome segregation’ and ‘nucleotide excision repair’ were enriched in AMSC specific subpopulations, indicating their high proliferative ability (Figure S19). The distinct PPIs among intra‐tissue subpopulations were also surveyed. The PPI analysis on DEGs in each subpopulation within each tissue was performed. The results showed that the different protein complexes were mainly enriched in specific subpopulations in each tissue (Figures S15D, S16D, S17C and S18D). Besides some complexes shared by two tissue‐derived MSCs, multiple complexes had tissue‐specific PPIs. For instance, the terms ‘antigen process and presentation of exogenous peptide antigen via MHC class II’ and ‘hematopoietic stem cell differentiation’ were only enriched in BMSC subpopulations (Figure S16D). In conclusion, the heterogeneous PPIs and transcriptomic regulons reflect the distinct biological functions in inter‐ and intra‐tissue MSCs.

### 3.8 Heterogenous trajectories of MSCs across multiple tissue origins

RNA velocity^28^ and trajectory analysis were performed on the unbiased clustering for each tissue to further assess the distinct developmental progression and resultant changes in gene expression across different types of MSCs. The potential directionality of cell state transitions was observed in BMSC and UMSC intra‐subpopulations, using an arrow indicating the direction of differentiation.^97^ This indicated that these subpopulations recapitulated early‐to‐late transition. However, no significant flow appeared within each of the two AMSC and DMSC subpopulations, revealing that these subpopulations were relatively stable and independent from each other (Figure S20A–D). Similarly, MSC subpopulations from umbilical cords exhibited the linear developmental progression without branch across pseudotime axis after aligning and reconstructing cells from each cluster using Monocle .^26^ In contrast, MSC subpopulations from the other tissues showed no linear distribution with branches (Figure S20E). Inter‐donor trajectories also showed that UMSCs had higher inter‐donor heterogeneity than the other tissue‐origins, consistent with the observation mentioned above (Figure 6A). Besides UMSCs, BMSCs also exhibited heterogeneous inter‐donor trajectories, especially between donor B01 and two others (B02 and B03) (Figure S14B).

**FIGURE 8:**
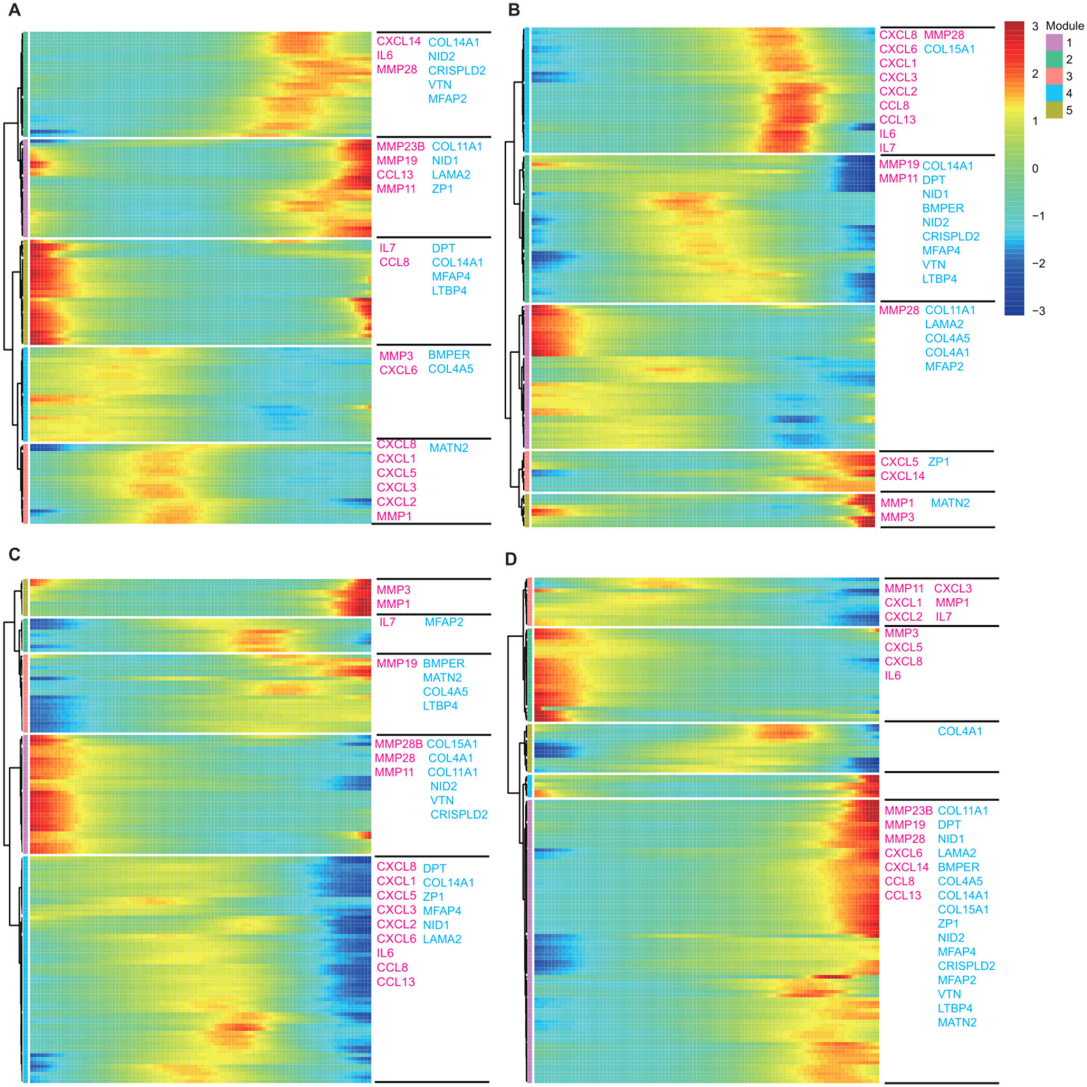
Gene dynamics of extracellular matrix (ECM) and pro‐inflammatory genes associated with inter‐donor heterogeneity. Heatmap depicting the expression of ECM (pink) and pro‐inflammatory (blue) genes along with temporal distribution in pseudotime for adipose (A), bone marrow (B), dermis (C) and umbilical cord (D), respectively. Genes were hierarchically clustered based on a pseudotime‐dependent expression. The genes were scaled as the percentage of maximum expression

The representative ECM‐associated genes within HVGs were used to assess whether the relationship between the expression of ECM‐associated genes across pseudotime have tissue‐specific heterogeneous. The dynamics of these ECM‐associated genes within HVGs exhibited alternative trajectories across tissues (Figure 8). Notably, these inter‐donor variable ECM genes were mainly co‐expressed in UMSCs at late pseudotime and were distinguished from other tissues derived from MSCs. This indicated that ECM alteration promotes UMSC heterogeneity thus decreasing UMSC stemness (Figure 8D). Furthermore, many pro‐inflammatory cytokines were co‐expressed along a trajectory in each tissue. CXCL1, CXCL2 and CXCL3 occurred synchronously in four types of MSCs (Figure 8).

ECM regulates MSC secretion relevant to hematopoietic recovery and angiogenesis.^98^ Pseudotime graph ordering was performed to examine the relative changes in many representative genes relevant to hematopoietic recovery and angiogenesis over pseudotime. The results that the different genes were clustered across multiple tissues along the pseudotime axis (Figure S21A–D). The expression of some genes, such as CCL2, exhibited a slight ‘ups and downs’ trend, which was similar in four types of MSCs (Figure S21E). However, the expression of pro‐hematopoietic factor CXCL12^99^ was significantly increased over pseudotime in UMSCs. CXCL12 expression peaked in DMSCs and BMSCs, or gully distribution in AMSCs (Figure S21F).

## 4 DISCUSSION

Due to heterogeneity and variation in the use of MSCs, clinicians, biologists and scientific societies are yet to reach a consensus regarding their biological functions or potential therapeutic uses.^100^ Heterogeneity of MSCs at multiple levels (including donors, tissue sources, subpopulations and individual cells) remains to be completely elucidated. More evidence indicated that culture parameters, such as medium, culture environment, and storage, greatly impact influence the phenotype and function of MSCs.^2^ The batch effect (described as technical variations caused by handling distinct batches of samples) has further been reported during scRNA‐seq library preparation. The present study maximumly reduced the experimental batch effect, and all of 11 samples under the same culture condition (even the same bottle for serum and cytokine) were maintained and performed FACs sorting & scRNA‐seq library construction simultaneously. The established comprehensive atlas of > 130 000 single‐MSC transcriptomes across four types of medical‐utilising tissue resources (derived from 11 donors) provides a rich resource to explore for investigating the multi‐dimensional characterisation of MSCs. The present work demonstrated some novel insights in various aspects.

First, signature genes of the identified tissue‐specific and conserved subpopulations were characterised, respectively (Figure 1D,E). Of note, numerous genes categorised into TFs, ligands, ligand‐binding receptors, and cytokines, were highly expressed in tissue‐specific subpopulations (Figure S2). BMSC‐specific C0 subpopulation predominantly expressed classic MSC marker genes, such as LEPR, CXCL12, and CXCL16. Many TFs, including SIM1, TFAP2A, TBX5, and HOXA13 were specifically expressed in the DMSC‐specific C5 subpopulation (Figure S2). However, the functions of the co‐expressed TFs remain to be determined.

Second, ECM highly contributed to MSC heterogeneity. Different from previous evidence on several hundreds of MSCs,^6^ the massive single‐cell transcriptomes, herein, revealed that distinct cell cycle does not play a major part in the heterogeneity of MSCs (Figure S3). Canonical pathway analysis of highly variable signature genes demonstrated that these genes are mainly functionally enriched in ECM‐associated terms, including ECM regulators and glycoproteins (Figure 2E). In particular, the diversity of these gene expression values exhibited more variable in tissue‐specific compared to conserved subpopulations (Figure 3A‐B, Figure S4D). In addition to the roles in determining stem cell lineages, ECM also regulate MSCs secretion which may ameliorate or reduce their immunosuppressive function.^67^ Additionally, the disturbance of ECM also affects the integrity of the stem cell niche, finally resulting in senescence‐associated phenotypes.^102^ However, the underlying mechanisms remain elusive.

Third, UMSC specific subpopulations have several undeniable therapeutic advantages. Previous reports showed that functional contradictory secretions of MSCs can promote immunosuppression or boost immune responses.^103^, ^104^ Herein, UMSC‐specific C4 was found to express lower levels of inflammatory factors and higher levels of several anti‐inflammation factors compared to other MSC subpopulations (Figure 3C,D). UMSC‐specific C4 MSCs induce MHC‐class II‐mediated antigen processing and presentation to activate immune responses.^105^ Through exploration of the expression of signature genes and pathway enrichment analysis, UMSC‐specific C4 and two conserved subpopulations (C1 or C8) were found to lack antigen processing and presentation capability (Figure 4, Figure S11). While low expression of ribosome genes, such as in the conserved subpopulation C8, potentially reduced stemness the capacity of stemness.^106^ It is of particular note that UMSC‐specific C4 exhibited a dominant role in anti‐ageing (Figure 5). A previous study demonstrated that low expression of inflammatory factors, such as in UMSC‐specific C4, also limits SASP,^78^ potentially preserving the functions of MSCs. Similarly, the current novel procedure for UMSCs successfully preserves the immunosuppressive and highly proliferative properties, potentially through reducing replicative senescence during in vitro expansion.^107^ Late‐passage (*p* > 10) or senescent MSCs correlate with the accumulation of the pro‐inflammatory signatures, thus compromising their immunomodulatory capacity.^108^ Thus, a better understanding of the interplay between inflammatory signatures and ageing of MSCs can facilitate the quality control of MSC‐based products for clinical use. In particular, the combination of functional selection based on MSC subpopulations and special in‐vitro procedures primarily^107^ improves the MSC‐based therapies.

Fourth, conserved and diverse inter‐tissue transcriptome regulons and PPIs (Figure 6) suggested common or tissue‐specific diverse functions of MSCs. Compelling evidence indicates that common regulons (including STAT3,^109^ STAT1,^110^ JUN^111^ and YBX1)^112^ in all tissue resources, exert crucial immunosuppressive effects. Additionally, BRCA1, TP53 and MYC maintain genomic stability and self‐renewals. The present work revealed numerous significantly activated regulons that exhibited tissue‐specificity. For example, CEBPB, PPARG, ETS2 and RUNX2 in AMSCs and TWIST1, RUNX3 and WT1 in DMSCs were revealed as the regulators of MSC lineage differentiation. These activated regulons also demonstrated intra‐tissue heterogeneity (Figure 7B–E). PPIs also exhibited their inter‐ or intra‐tissue heterogeneity. For example, subpopulations of BMSCs featured as the function of antigen processing and presentation or hematopoietic stem cell differentiation (Figure S15). Of note, several signature genes of each subpopulation were clustered in the same functional terms (Figure S15D, S16D and S17C), implying that they may perform similar functions when co‐expressed.^113^ In view of these findings, further studies are warranted to determine the functional roles of signature genes in MSCs.

Fifth, distinct donor variations of MSCs across multiple tissue sources have been resolved. There is evidence that highly plastic cells can give rise to diverse fates, including cancer cells and stem cells.^114^ During tumour evolution, abnormal somatic cells as mature terminal‐differentiated cells can possess stem‐like phenotypes under certain conditions. A reversible dedifferentiation of tumour cells exits varying degrees, thus contributing to tumour heterogeneity.^115^ Moreover, the cellular plasticity of adult stem cells is characteristics of self‐sustenance and differentiation into one or multiple lineages. Pieces of evidence from lineage‐tracing studies demonstrate the high heterogeneity of hematopoietic stem cells (HSCs) in capacities of self‐renewals and lineage‐biased capacities.^116^ In a hematopoietic hierarchy, evidence shows that diverse directions decreased from stem cells to progenitors, which progressively reduced phenotypic heterogeneity.^117^ Furthermore, deep learning analysis of cancer omics‐data also demonstrates that stemness indices are positively correlated with oncogenic dedifferentiation, reflecting tumour heterogeneity.^118^ The present investigation reveals UMSCs exhibited more donor‐to‐donor heterogeneity, particularly in ECM components and their related inflammation, antigen processing and ageing (Figures S8 and S9). Also, several lineage‐differentiation genes exhibit high donor‐heterogeneity in UMSCs (Figure S14A). Nevertheless, the global transcriptomic evaluation demonstrated high similarity across different donors from multiple tissue sources (Figure 6B). UMSC subpopulations exhibit a relatively near‐linear developmental progression, whereas the subpopulations from the other tissue resources show no linear distribution across the pseudotime axis, which provide evidence of more complicated trajectories (Figure S20). UMSCs exhibit larger donor‐variation over pseudotime compared to the other tissue resources (Figure S14B), which may be ascribed to higher primitivity of stem cells from perinatal tissues than other adult stem cells.^119^ These findings demonstrate that highly primitive stem cells are more heterogeneous. As such, our MSC atlas across different tissue resources provide more clues to an understanding of stem cell behaviours during tissue development.

Emerging evidence shows that accurate computational methods to remove doublets in scRNA‐seq data are lacking and current algorithms, including Doubletfinder^24^ and Scrublet.^120^ Scds^121^ or DoubletDecon^122^ may only computationally predict the doublets or artefacts occurring between distinct cell types (heterotypic doublets or multiplets). As such, it is possible to achieve doublet/multiplet removal experimentally via cell hashing^123^ (pooling of multiple samples labelled with distinct oligo‐tagged antibodies) or through genotype‐based multiplexing, cellular barcoding,^124^ and so on. However, the purified MSCs (homotypic doublets or multiplets) in the present work are not feasible. Additionally, some methods require prior estimation of doublet or multiplet rate, which cannot be evaluated accurately and strongly exhibit strong subjective bias. Due to the scarcity of distinctive markers and similarity in morphology, MSCs are almost indistinguishable from fibroblasts,^125^ which remains a limiting factor for accurate functional studies of fibroblasts and MSCs. Consistent with MSCs, fibroblasts also contribute to tri‐lineage differentiation and immunosuppression. In addition, all fibroblasts, much like MSCs, are positive for CD73, CD90 and CD105 and negative for CD14, CD34, CD45, CD19, and HLA‐DR. Though some CD11b+ fibroblast subtypes exist in the kidney,^125^ CD11b is negative for all MSCs. In the recent past, fibroblasts were considered as aged MSCs.^126^ The cells we collected were defined as MSCs, according to the MSC criteria provided by the International Society for Cellular Therapy. Current evidence indicates that subtypes and markers of fibroblasts and MSCs are only present in mouse bone marrow. Notably, our dataset of MSCs from multiple tissues can support distinguishable molecular markers and phenotypes between human fibroblasts and MSCs in considered tissues in future work.

This study has some limitations. First, the scale of this research is limited in types of tissue resources and donor number for each tissue. The number of samples was too small to confidently eliminate unknown confounding factors similar to many single‐cell transcriptomic atlases. The design of the current analysis only focussed on the most but not all frequently used MSCs in clinical trials. We made the utmost attempt to reduce the experimental batch effect despite the limited number of donors for each tissue. However, the heterogeneity here was shown to be the greatest between cells from different sources. Collectively, these observations bring future opportunities for an all‐encompassing MSC Atlas in all human tissues and a huge number of donors. Second, some donor sources are sex‐dependent. For instance, the foreskin is specific to males, and the umbilical cord is specific to females. As such, overexpression and knockdown of sex‐dependent genes in the same donor‐derived MSCs would be a good choice in the future to better exclude donor‐to‐donor variations. Third, this work mainly explores the public MSC atlas and defines tissue‐specialised subtypes. We acknowledge that functional validation, especially in the mouse model, is required to verify the putative role of each phenotype of the MSC cluster. The phenotypes of each MSC cluster are only based on transcriptomic data. Last but not least, consistent with many single‐cell transcriptomic atlases on solid tissues, we could not ignore the possibility that certain subpopulations were possibly lost during the isolation and FACS‐sorting enrichment, or clonal expansion in vitro. Nonetheless, these potentially lost subpopulations may be lack of utilisation in current clinical trials.

## 5 CONCLUSION

MSC atlas taxonomy in health conditions first systematically throws light on inter‐ and intra‐tissue, and donor‐to‐donor heterogeneity at the single‐cell level under the consistent system. This MSC census provides deeper insight to understand MSC immunosuppressive uniformity and heterogeneity. Furthermore, the findings offer a valuable resource for exploring multiple functions and underlying mechanisms of MSCs, therefore, has broad implications for MSC basic research and clinical applications.

## CONFLICT OF INTEREST

The authors declare no conflict of interest.

## Supporting information



Supporting Information

Supporting Information

Supporting Information

Supporting Information

Supporting Information

Supporting Information

Supporting Information

Supporting Information

Supporting Information

Supporting Information

Supporting Information

Supporting Information

Supporting Information

Supporting Information

Supporting Information

Supporting Information

Supporting Information

Supporting Information

Supporting Information

Supporting Information

Supporting Information

Supporting Information

Supporting Information

Supporting Information

Supporting Information

Supporting Information

Supporting Information
