# Supplementary figures and images for: Single‐cell transcriptome atlas of human mesenchymal stem cells exploring cellular heterogeneity
Source: Clin Transl Med. 2021 Dec 29;11(12):e650. doi: 10.1002/ctm2.650 (PMC8715893; doi:10.1002/ctm2.650)

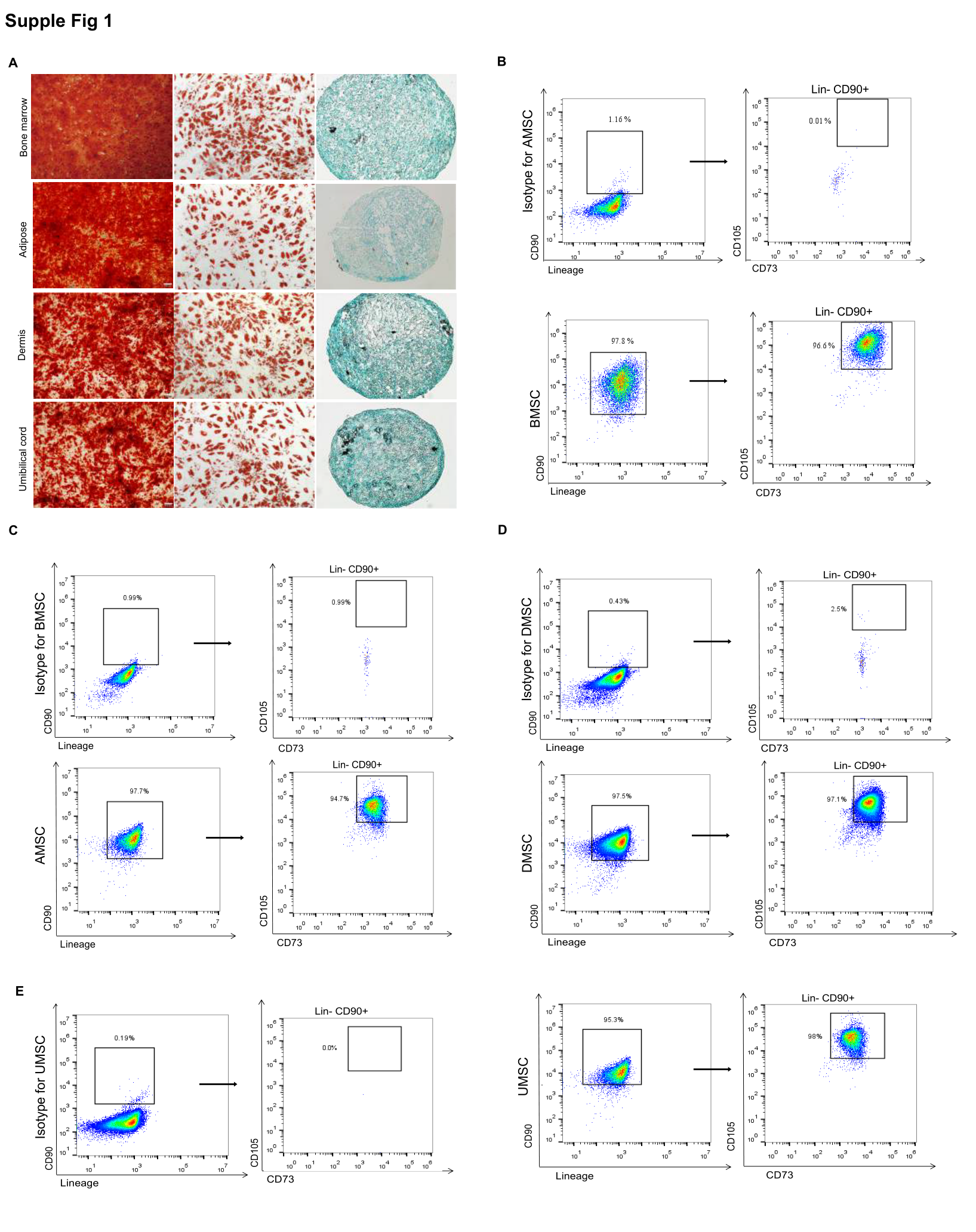

Supplement: Supplementary file 1 — Supporting Information [file CTM2-11-e650-s020.tif]

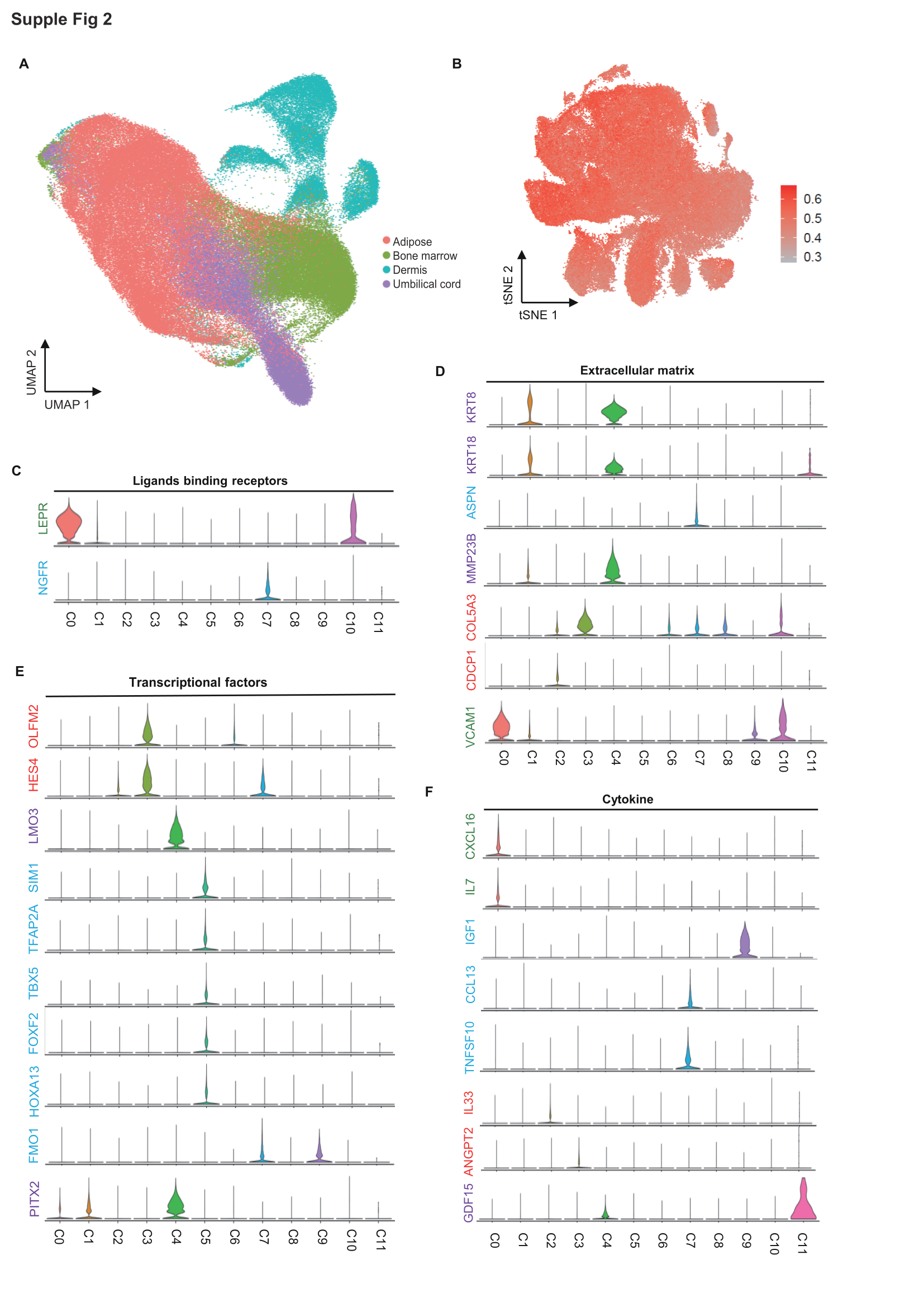

Supplement: Supplementary file 2 — Supporting Information [file CTM2-11-e650-s022.tif]

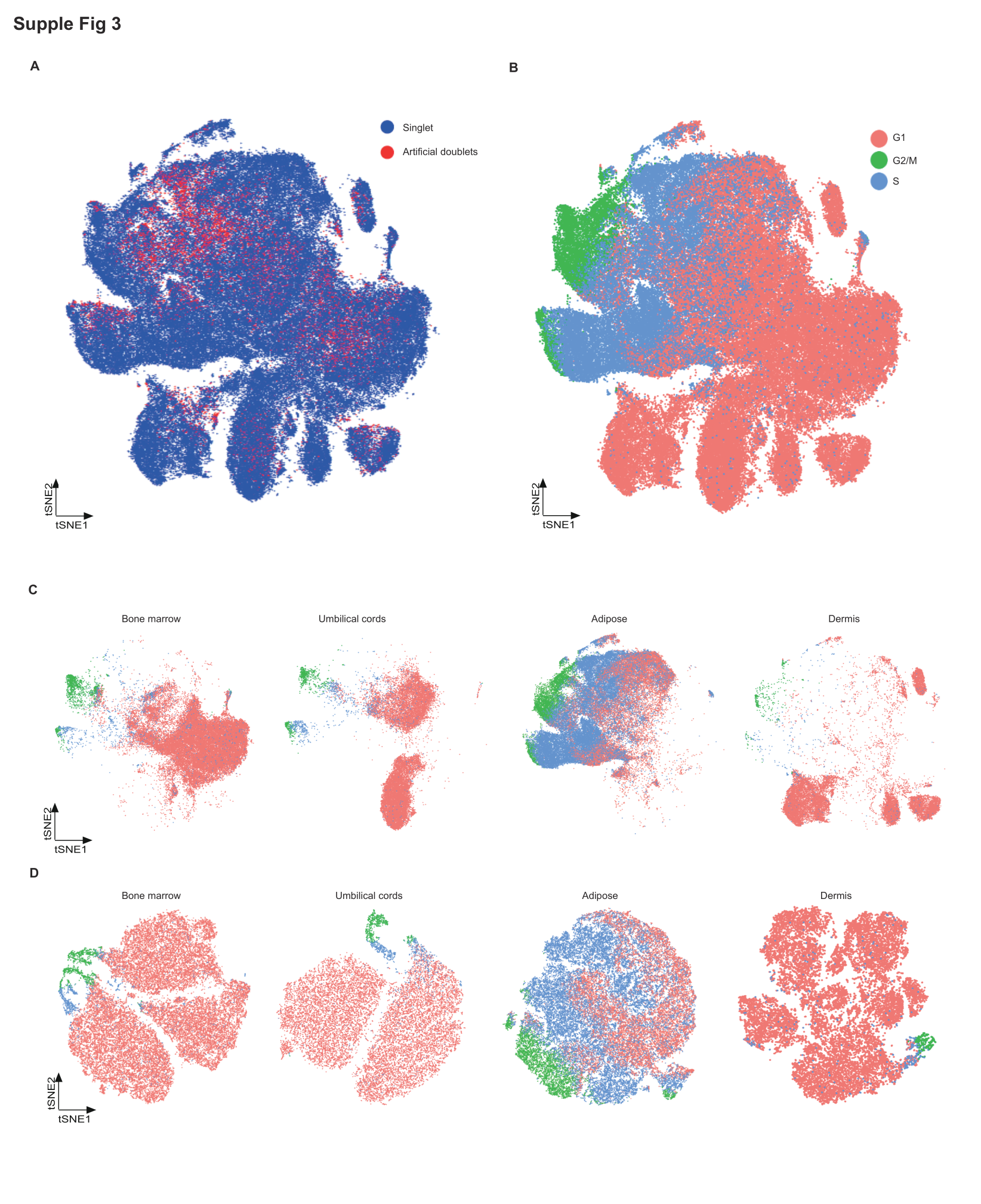

Supplement: Supplementary file 3 — Supporting Information [file CTM2-11-e650-s004.tif]

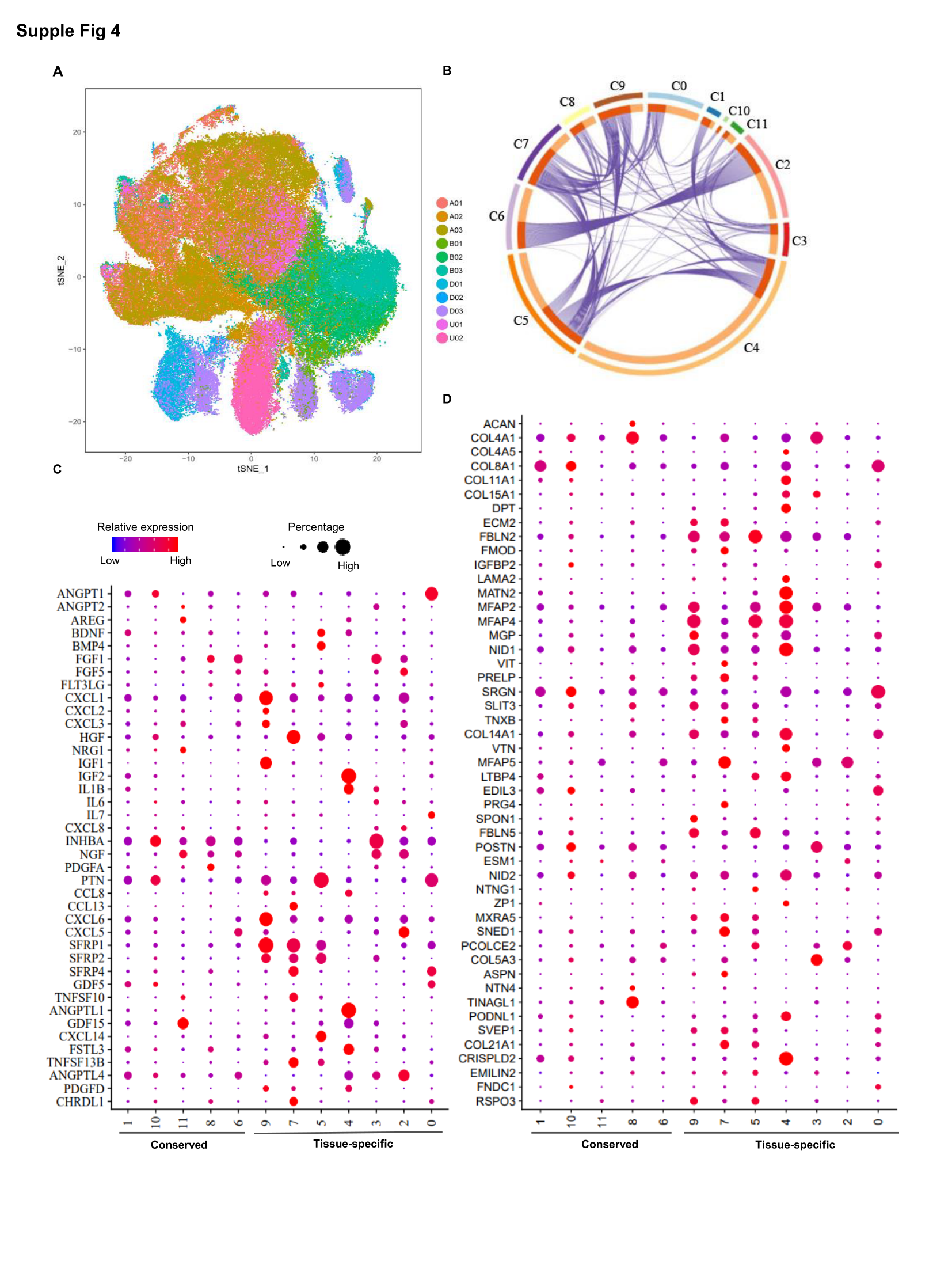

Supplement: Supplementary file 4 — Supporting Information [file CTM2-11-e650-s012.tif]

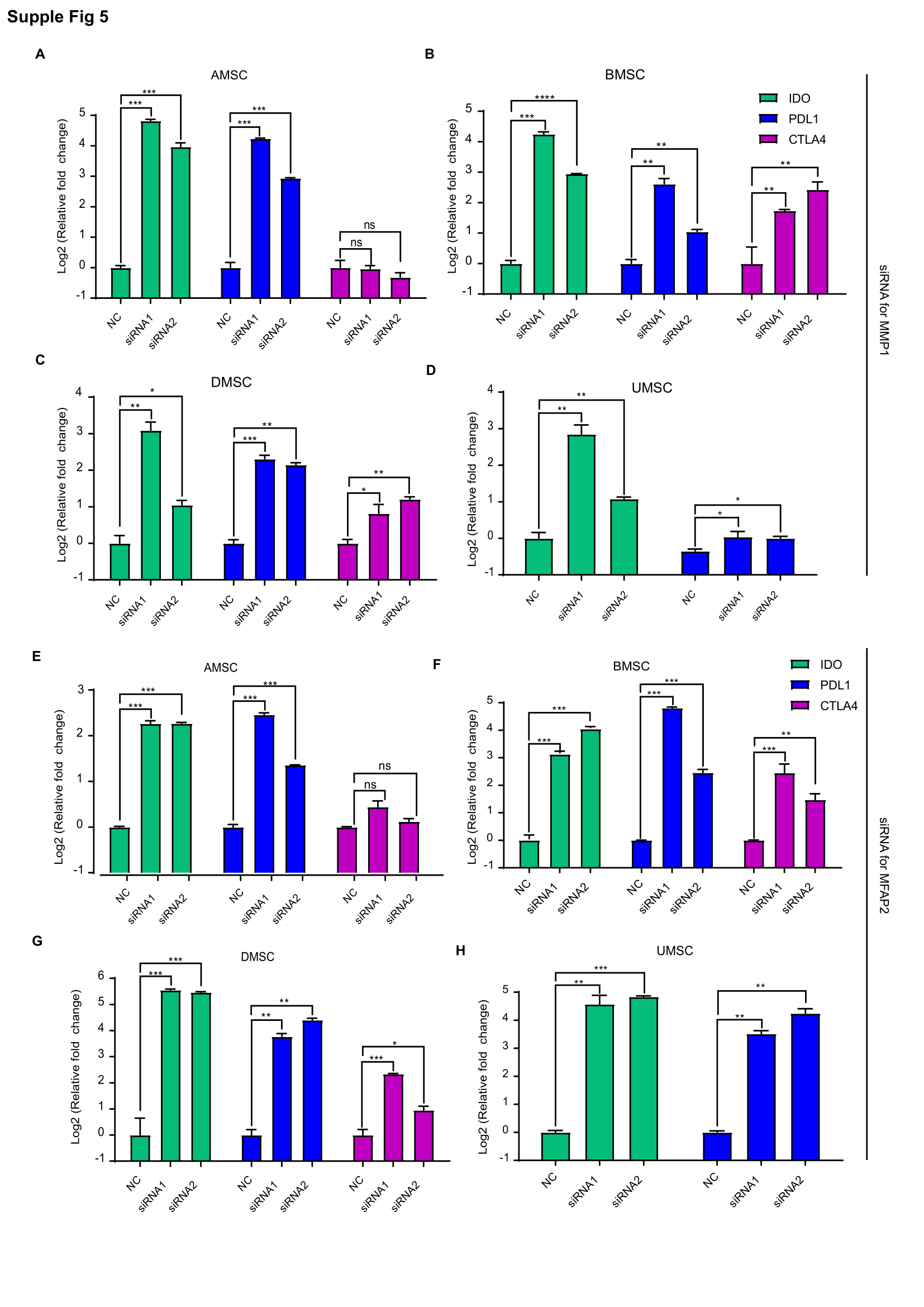

Supplement: Supplementary file 5 — Supporting Information [file CTM2-11-e650-s003.tif]

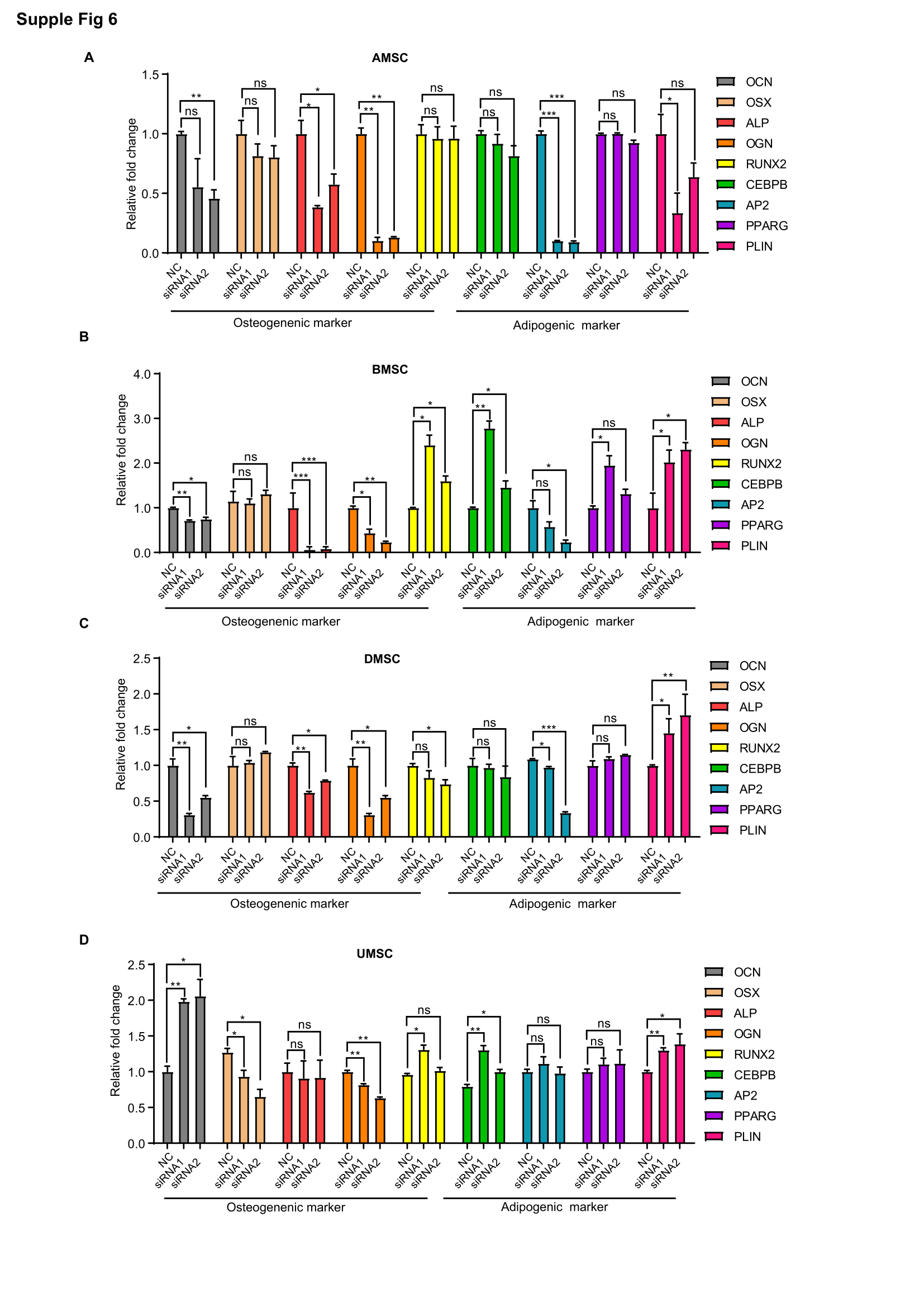

Supplement: Supplementary file 6 — Supporting Information [file CTM2-11-e650-s027.tif]

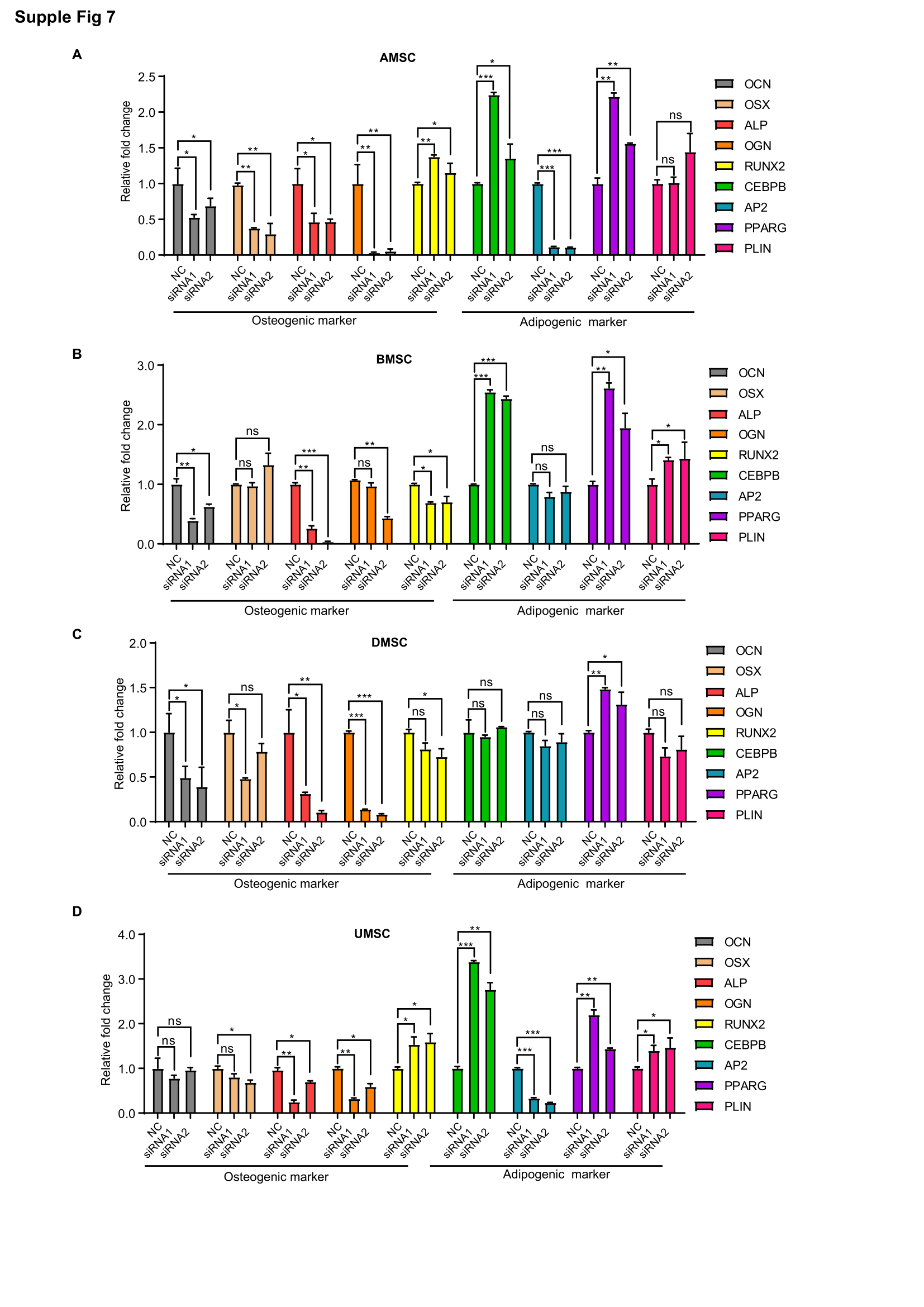

Supplement: Supplementary file 7 — Supporting Information [file CTM2-11-e650-s018.tif]

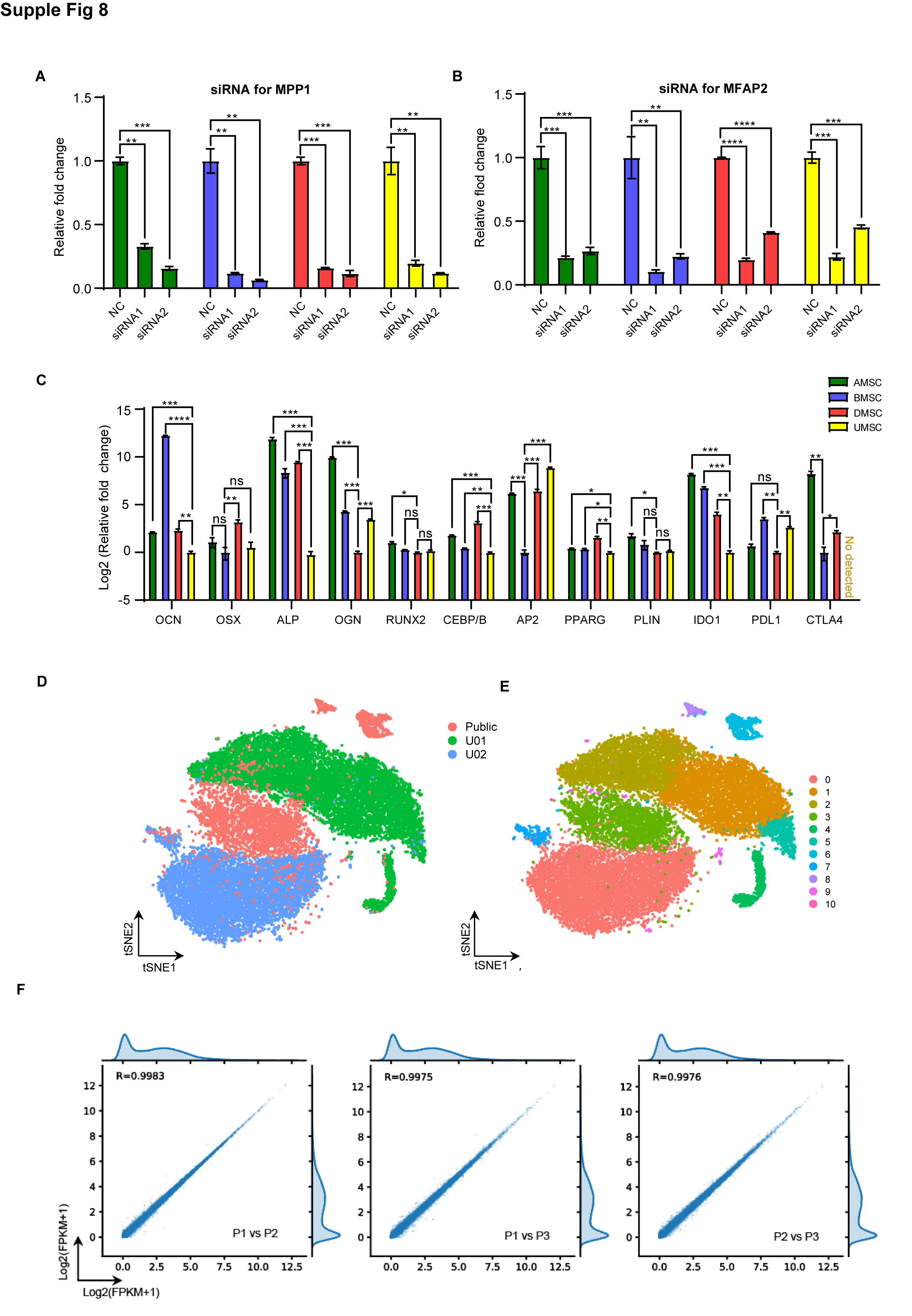

Supplement: Supplementary file 8 — Supporting Information [file CTM2-11-e650-s008.tif]

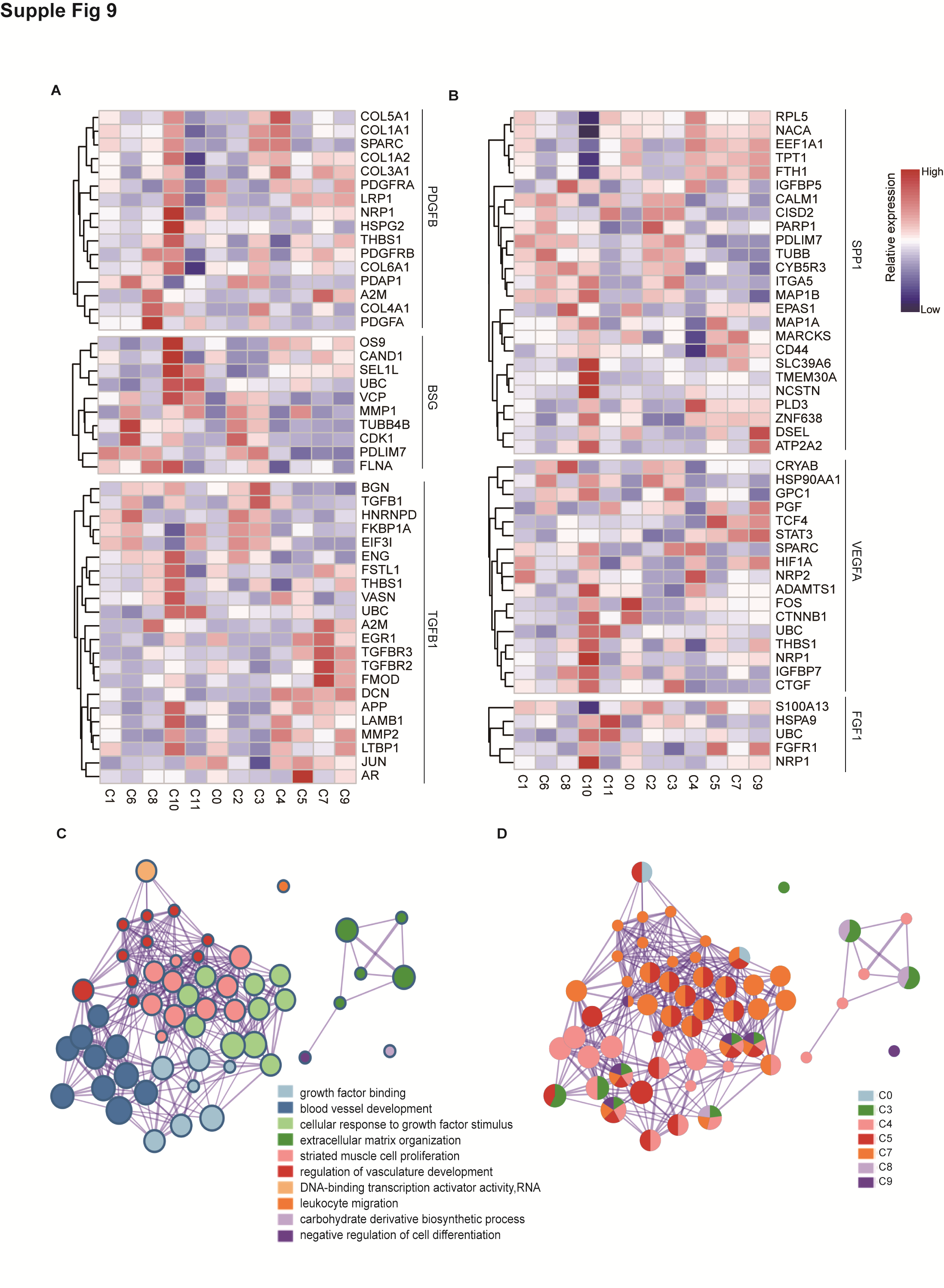

Supplement: Supplementary file 9 — Supporting Information [file CTM2-11-e650-s021.tif]

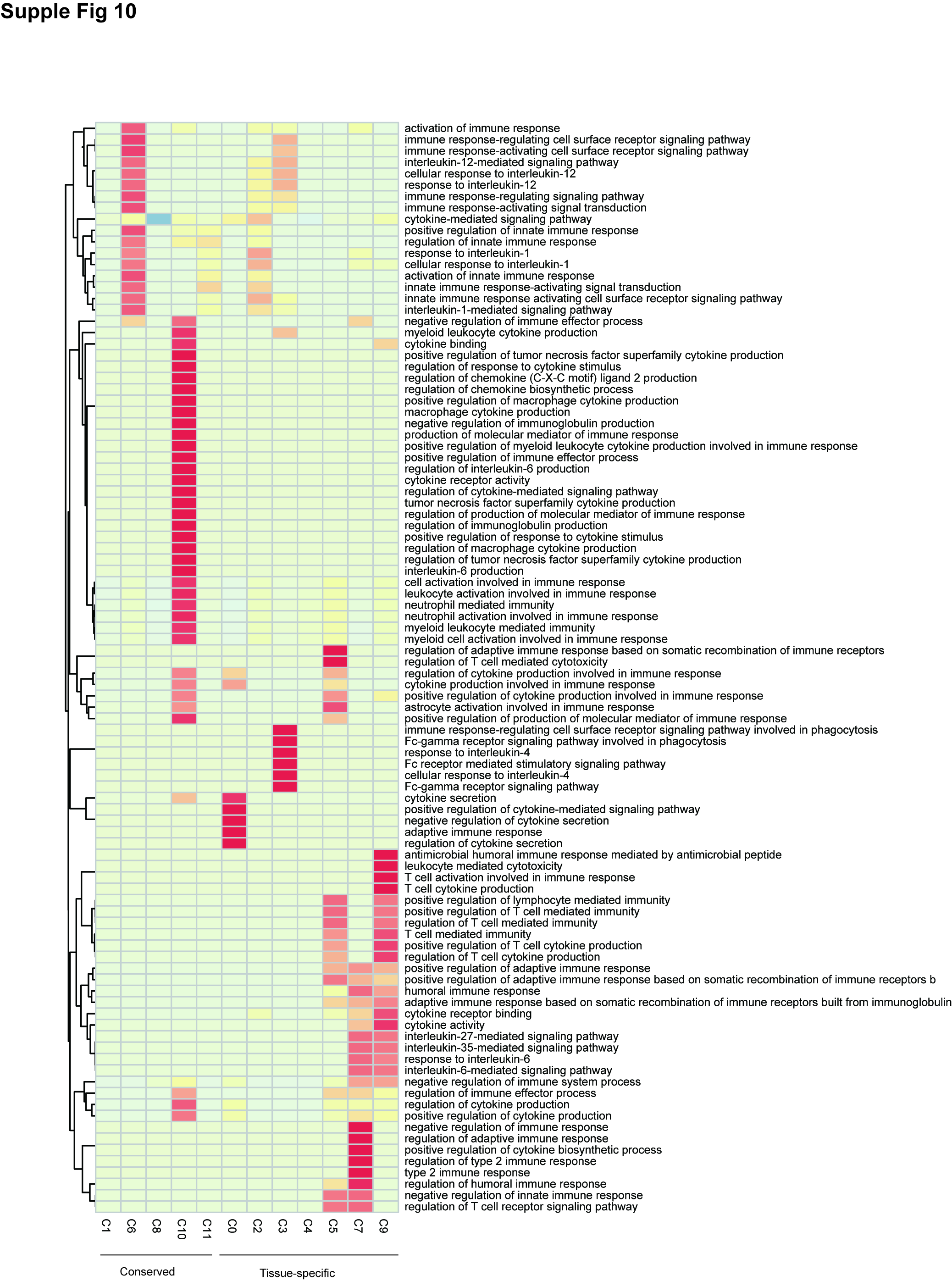

Supplement: Supplementary file 10 — Supporting Information [file CTM2-11-e650-s006.tif]

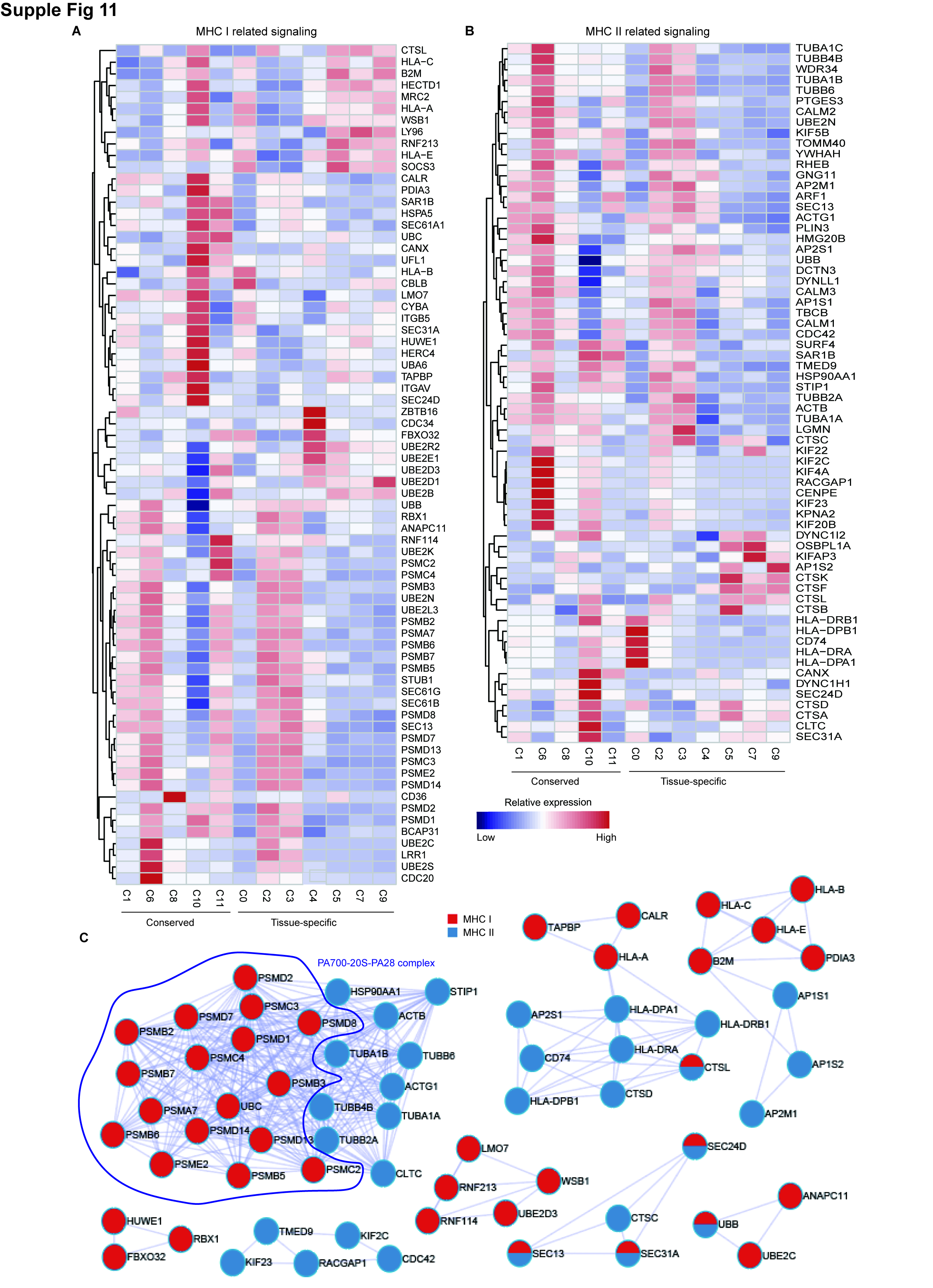

Supplement: Supplementary file 11 — Supporting Information [file CTM2-11-e650-s019.tif]

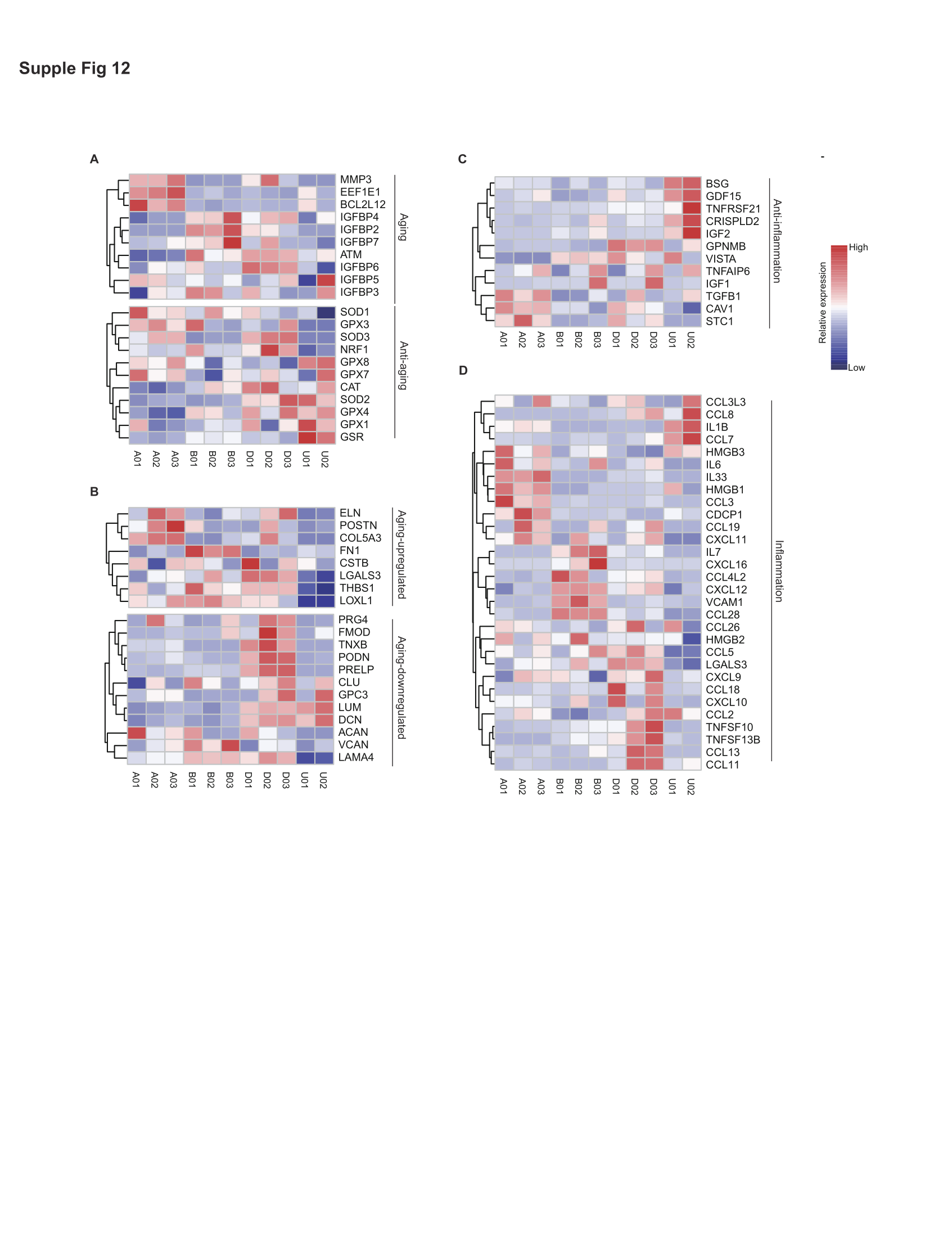

Supplement: Supplementary file 12 — Supporting Information [file CTM2-11-e650-s011.tif]

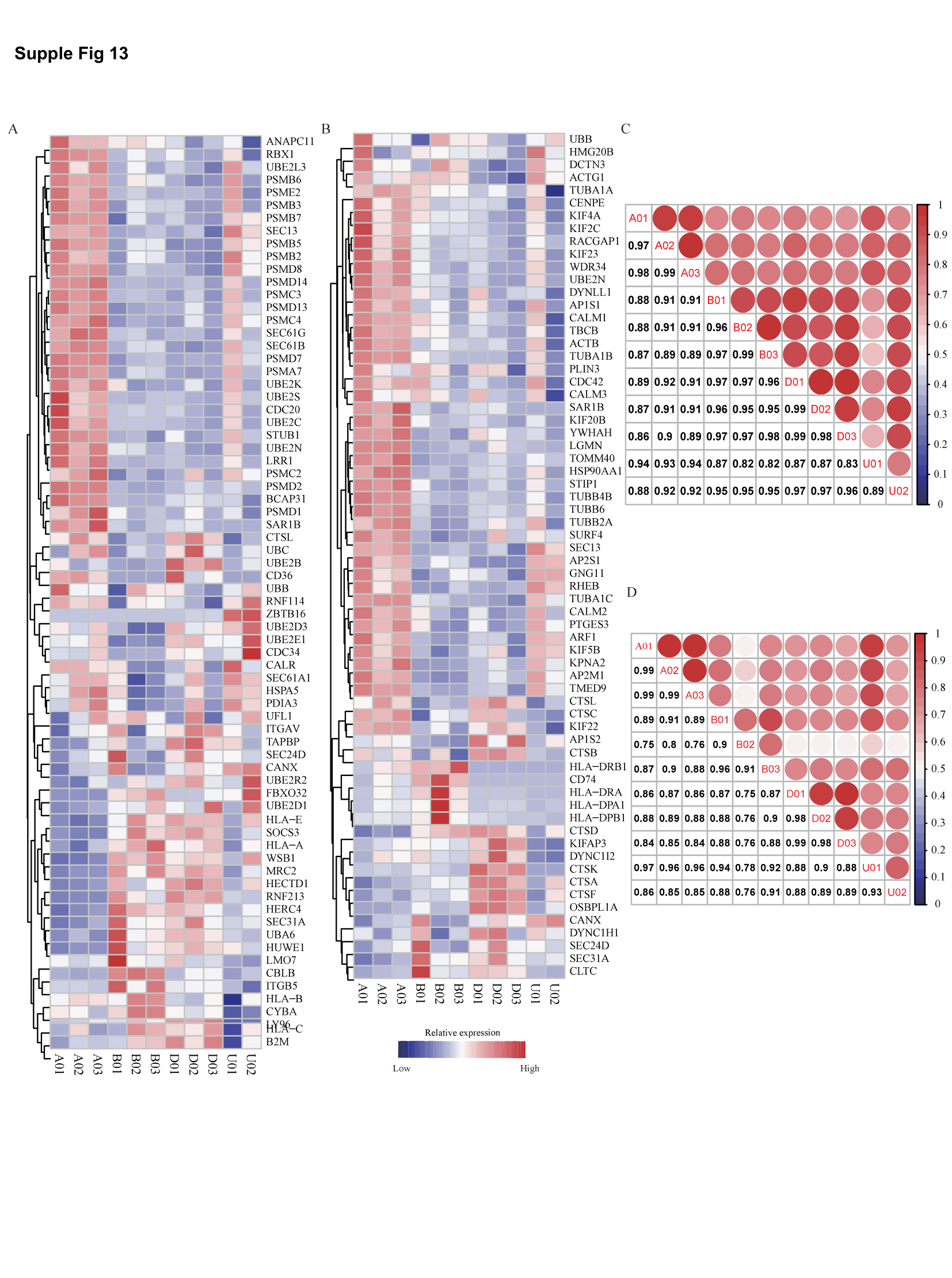

Supplement: Supplementary file 13 — Supporting Information [file CTM2-11-e650-s026.tif]

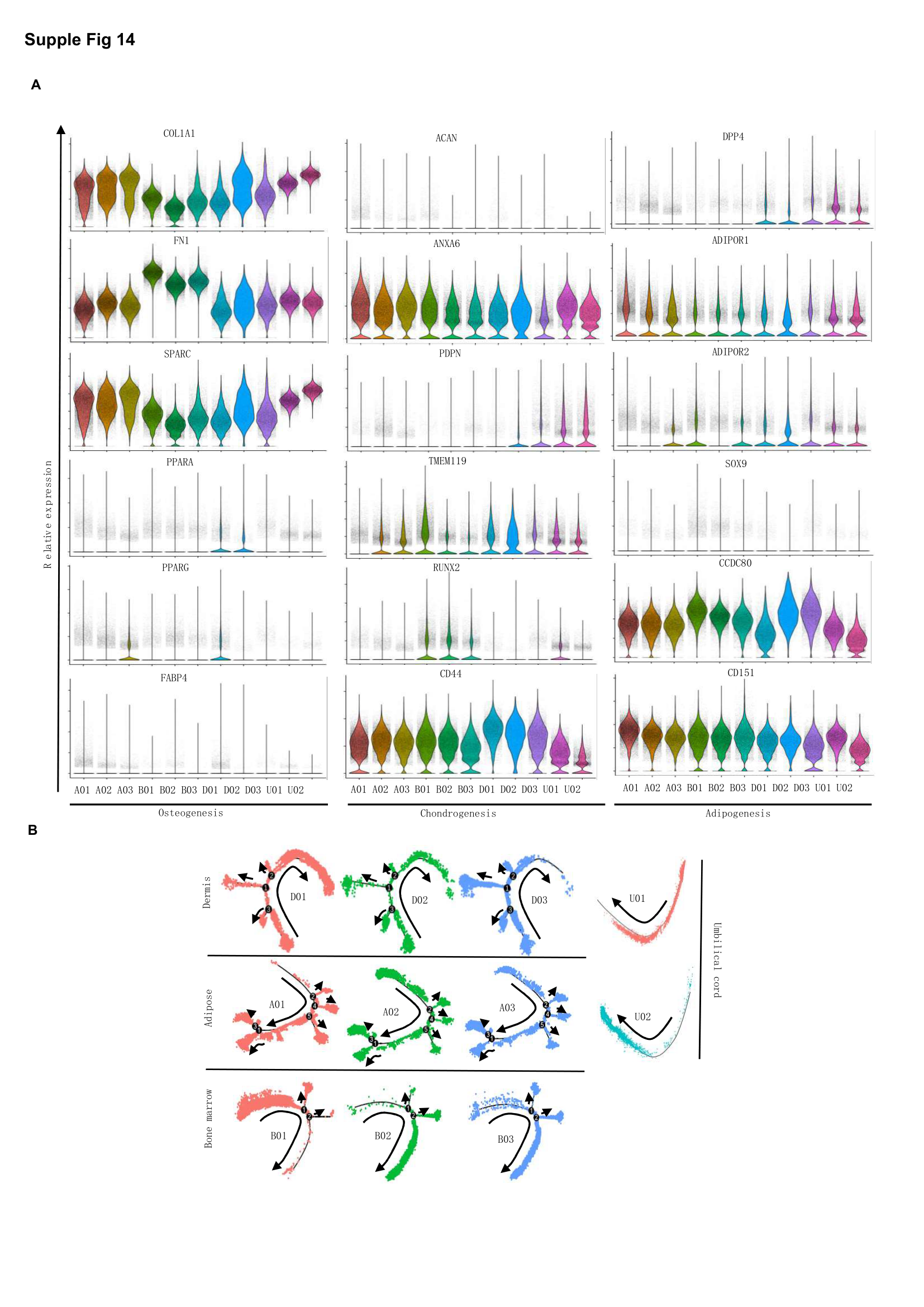

Supplement: Supplementary file 14 — Supporting Information [file CTM2-11-e650-s001.tif]

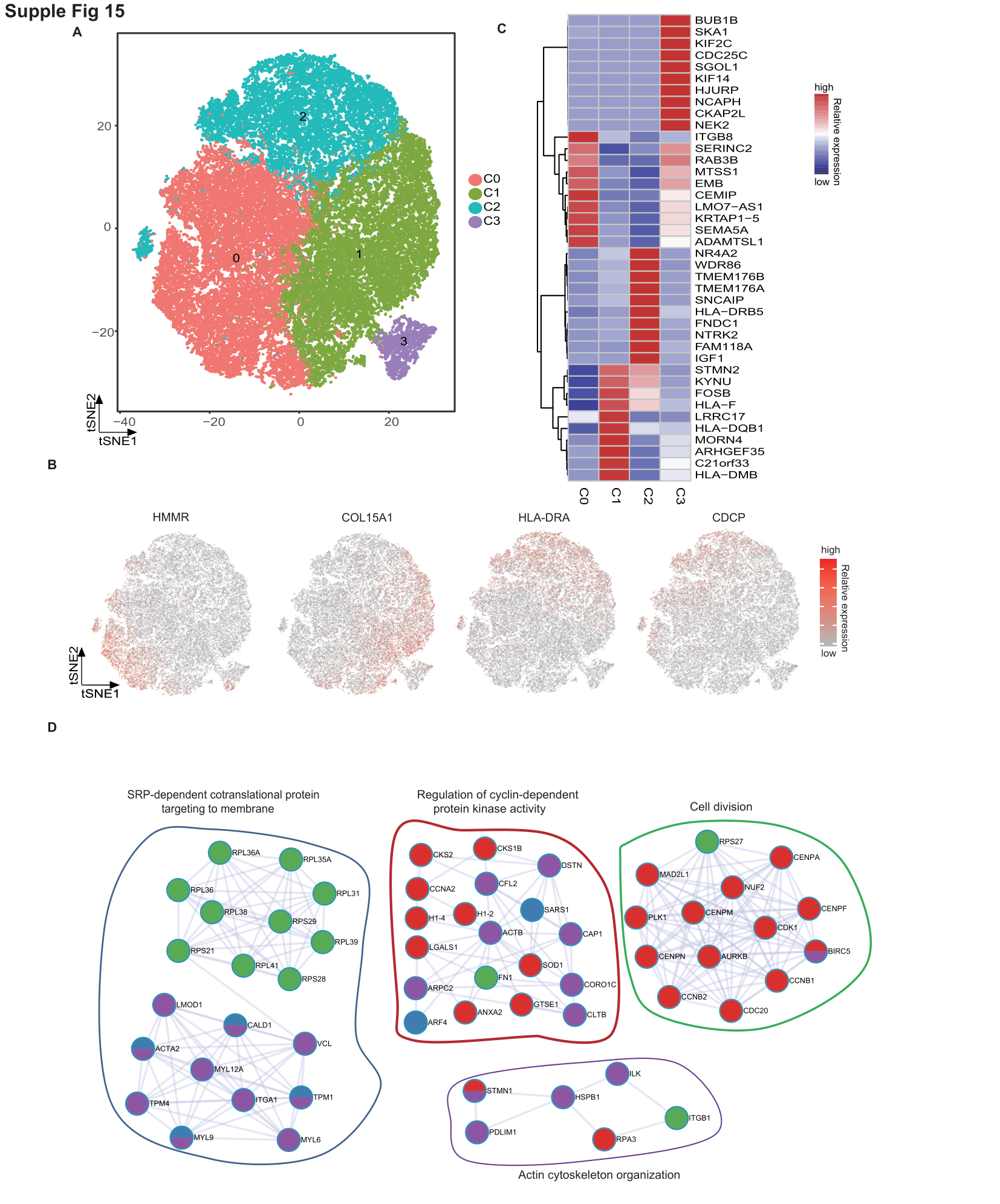

Supplement: Supplementary file 15 — Supporting Information [file CTM2-11-e650-s017.tif]

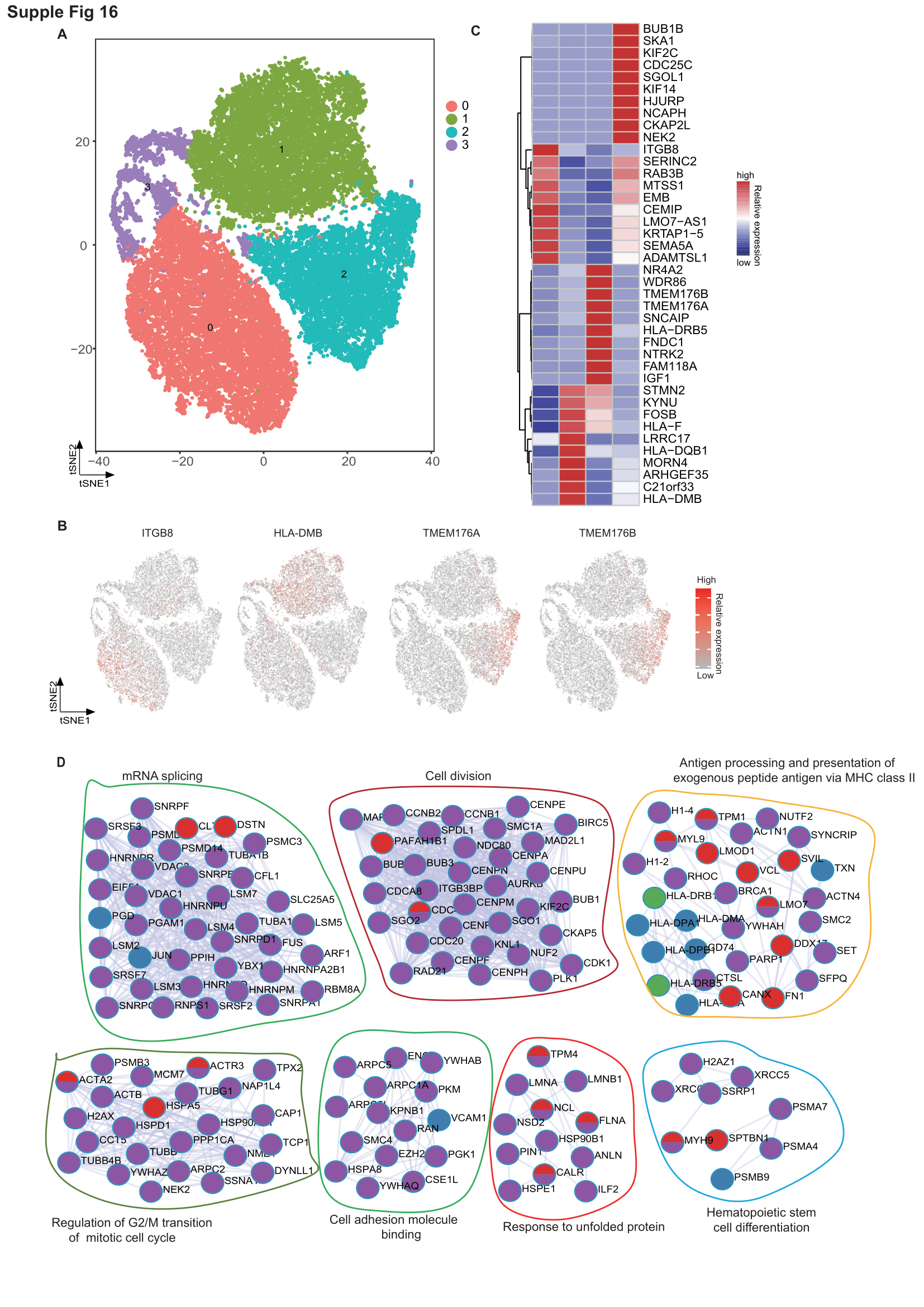

Supplement: Supplementary file 16 — Supporting Information [file CTM2-11-e650-s024.tif]

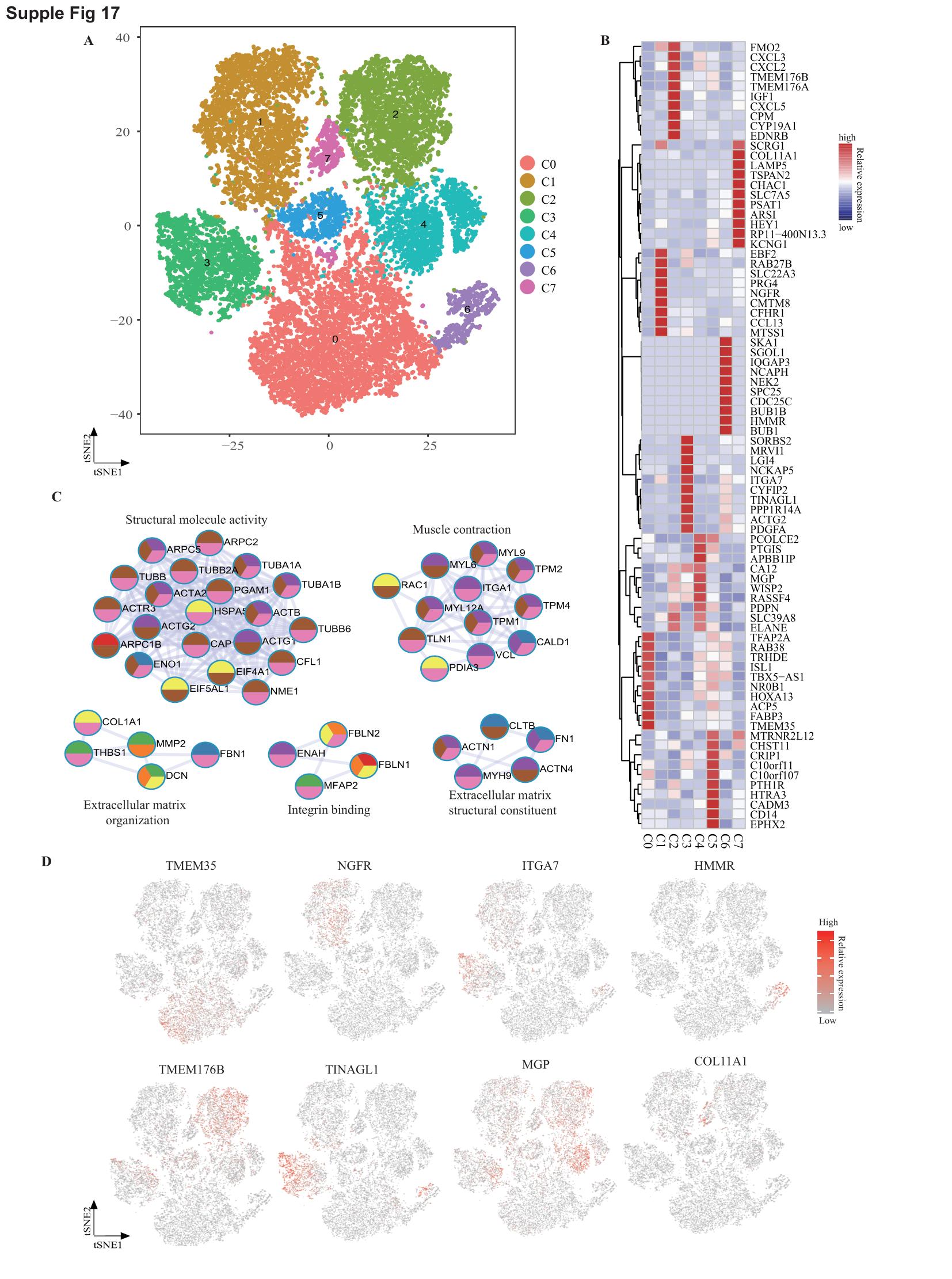

Supplement: Supplementary file 17 — Supporting Information [file CTM2-11-e650-s010.tif]

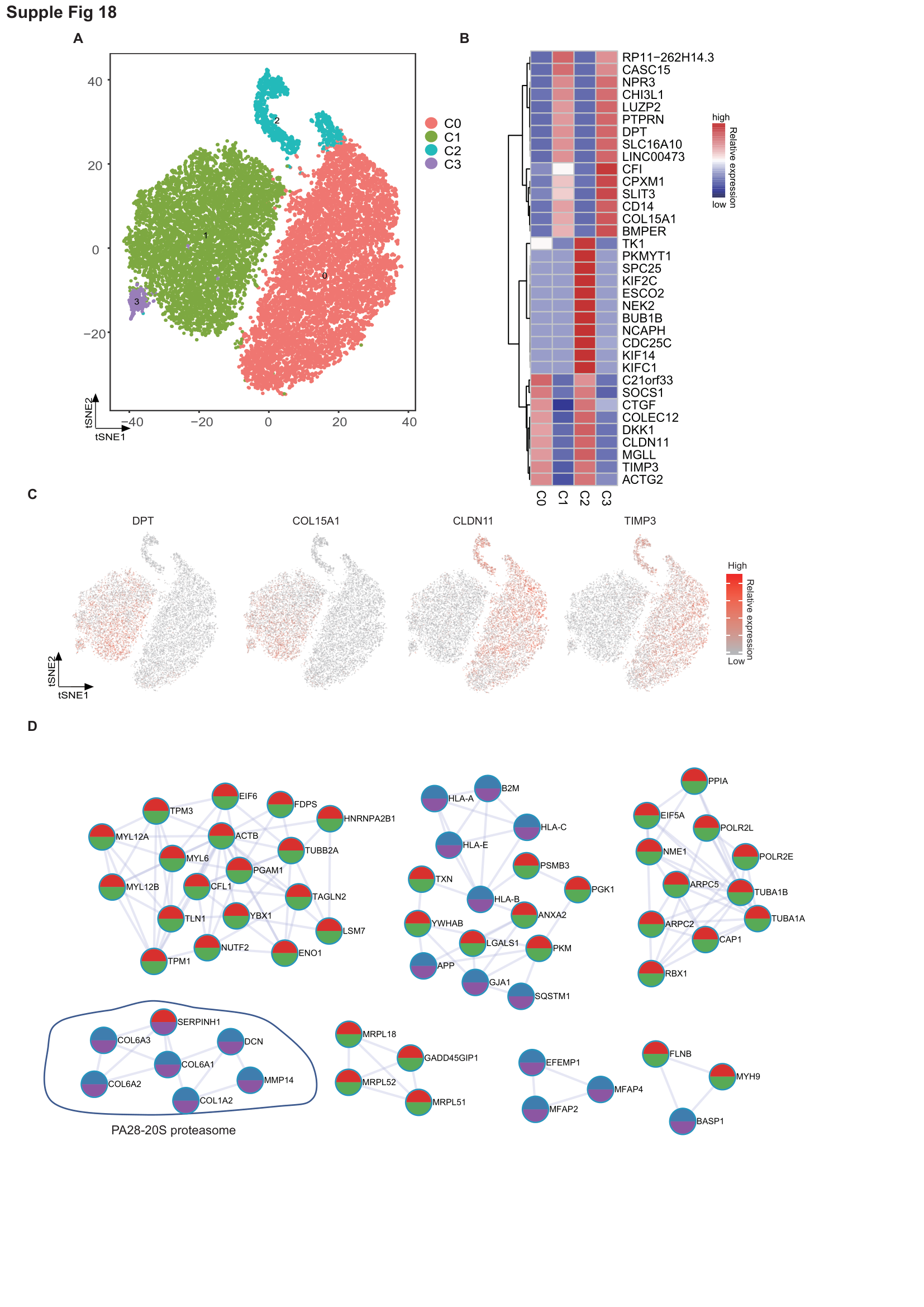

Supplement: Supplementary file 18 — Supporting Information [file CTM2-11-e650-s015.tif]

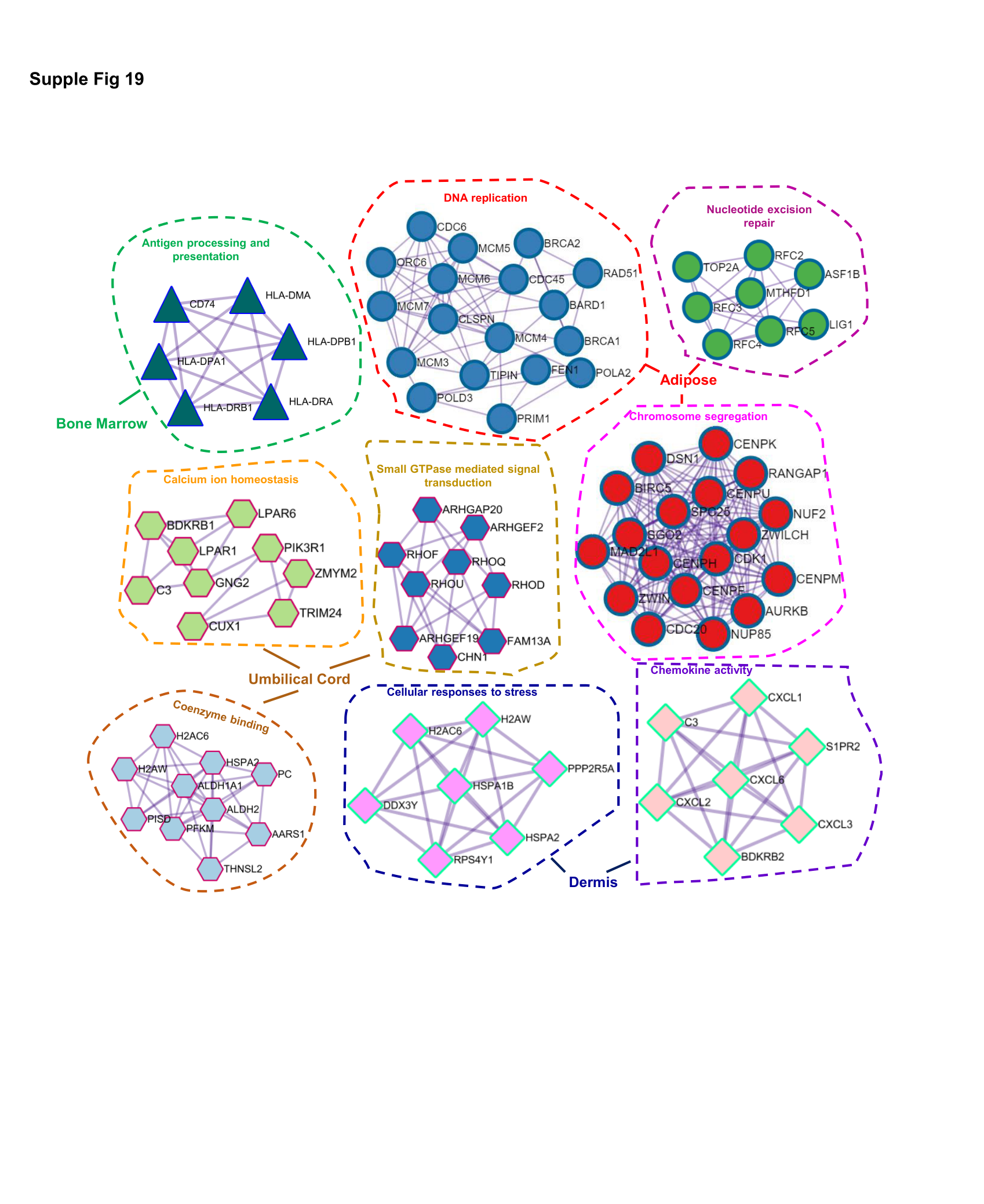

Supplement: Supplementary file 19 — Supporting Information [file CTM2-11-e650-s002.tif]

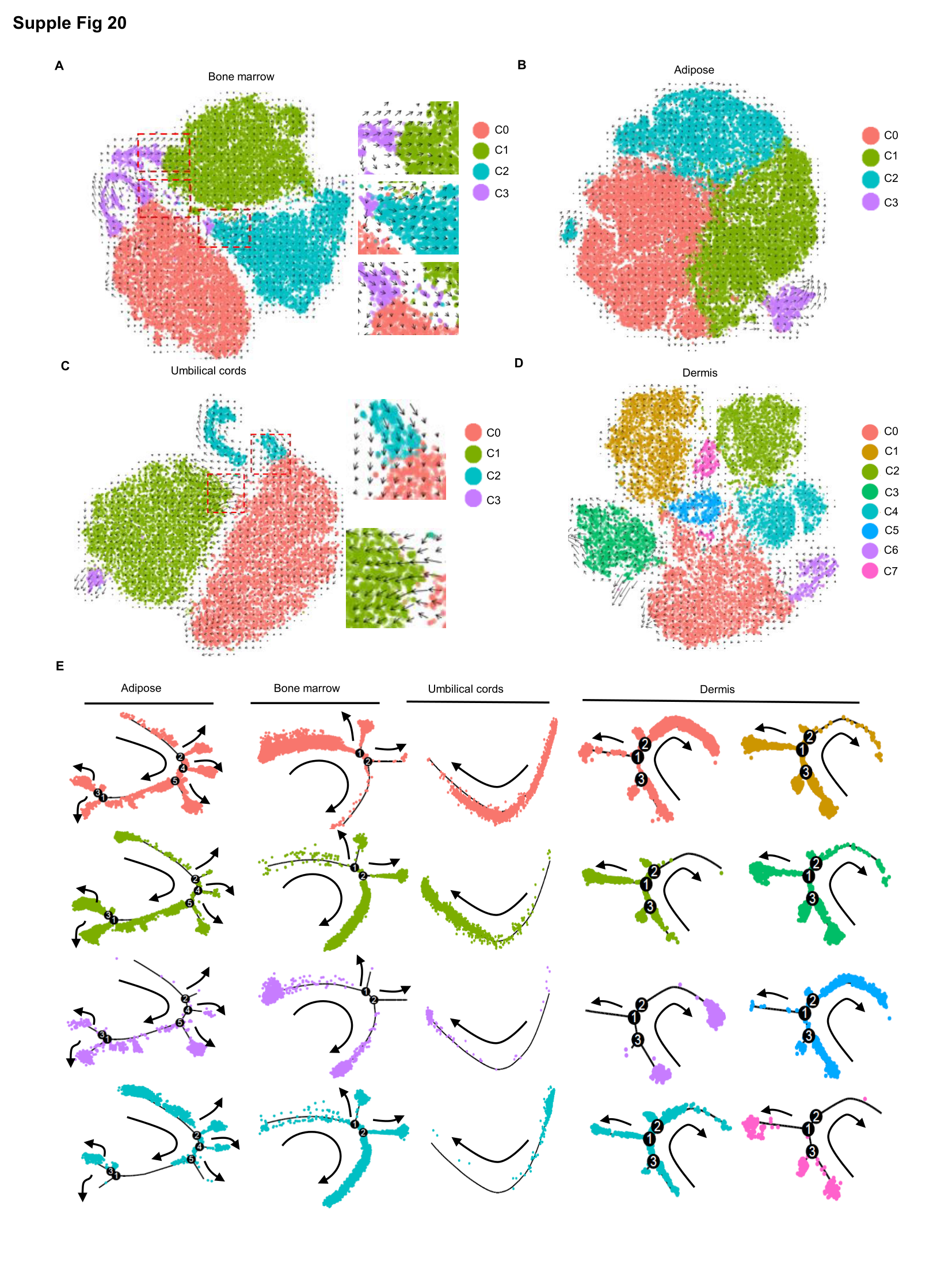

Supplement: Supplementary file 20 — Supporting Information [file CTM2-11-e650-s023.tif]

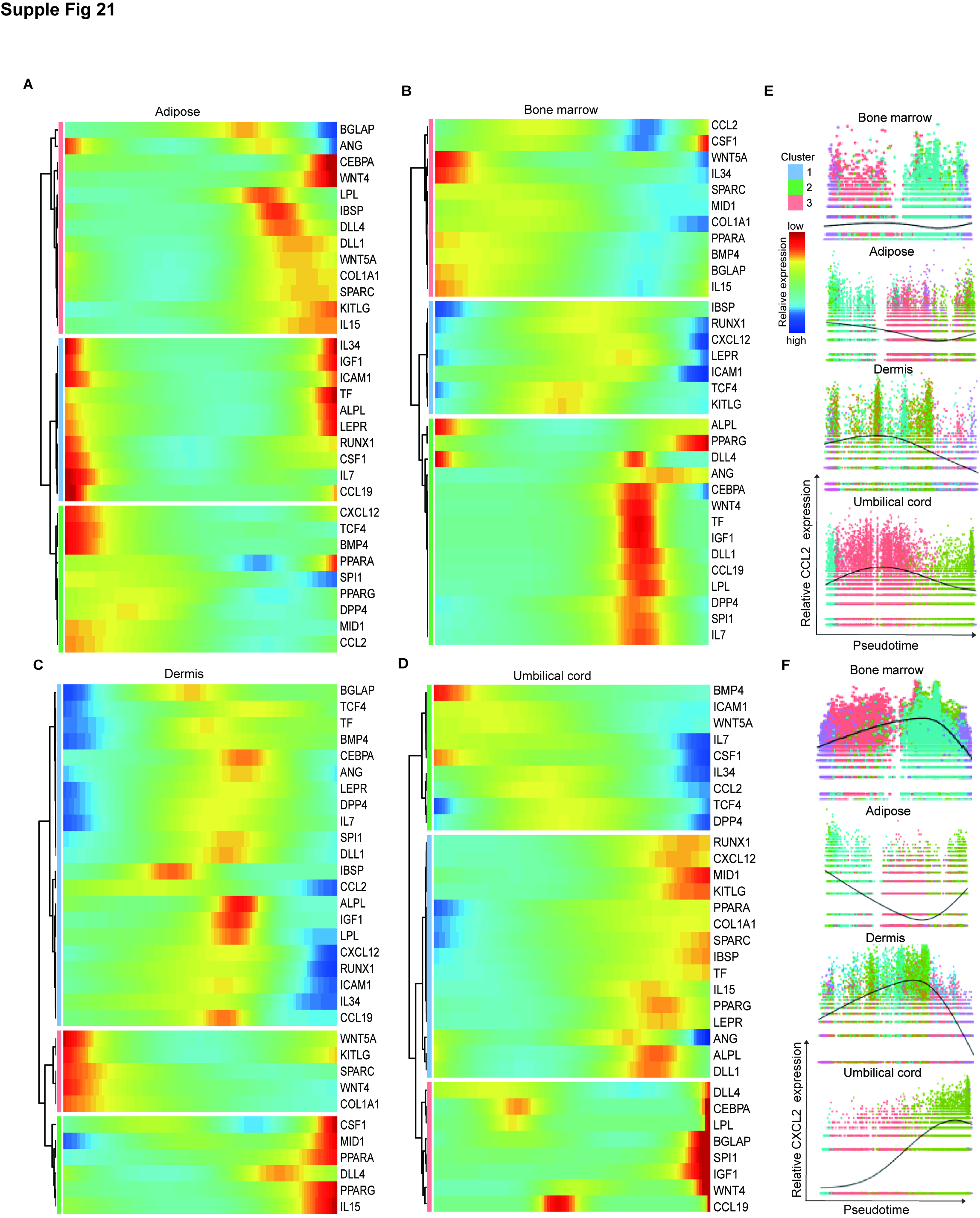

Supplement: Supplementary file 21 — Supporting Information [file CTM2-11-e650-s009.tif]
