# Supplementary material for: Single‐cell transcriptome atlas of human mesenchymal stem cells exploring cellular heterogeneity
Source: Clin Transl Med. 2021 Dec 29;11(12):e650. doi: 10.1002/ctm2.650 (PMC8715893; doi:10.1002/ctm2.650)
Supplement: Supplementary file 27 — Supporting Information [file CTM2-11-e650-s013.docx]

**Supplementary information**

**Single-Cell Transcriptome Atlas of Human Mesenchymal Stem Cells Uncovers Cellular Heterogeneity**

Zheng Wang^1,2#^, Chengyan Chai^1,2^, Rui Wang^1,2^, Yimei Feng^1,^^2^, Lei Huang^3^, Yiming Zhang^4^, Xia Xiao^5^, Shijie Yang^1,2^, Yunfang Zhang^1,2^, Xi Zhang^1,2#^

**SUPPLEMENTARY FIGURE LEGENDS**

**Figure S1.** **Quality control and identification of human isolated MSCs. (A)** Trilineage in vitro differentiation（osteogenesis, chondrogenesis and adipogenesis）of BMSCs, AMSCs, DMSCs and UMSCs was used to assess quality of isolated MSCs. Representative images for each tissue were shown for each tissue derived MSCs. Differentiated cells were identified by Alizarin-Red staining for osteoblasts, Oil-Red staining for adipocytes, and alcian blue staining for chondrocytes, respectively. Scale bar: 200 μm. **(B-E)** Representative flow cytometry histograms demonstrating MSC markers in populations of human isolated MSCs derived from adipose (**B**), bone marrow(**C**), dermis (**D**), and umbilical cord (**E**). The MSCs were positive for CD73, CD90, and CD105, and negative for hematopoietic lineage markers CD45, CD34, CD19, CD11b and HLA-DR.

**Figure S2. Tissue-specific marker genes of MSCs. (A**) UMAP plot of MSCs on all samples merged. color coded for tissue type. **(B)** Reference-based annotation of scRNA-seq using SingleR. t-SNE plot of spearsman correlation score for individual cells, colored scaled by red (high score) and gray (low score). Top 10 marker genes in tissue-specific clusters were enriched in ligand binding receptors **(C)**, extracellular matrix **(D)**, transcriptional factors **(E)** and cytokines **(F)**. Violin plot showing the relative expression. Colors representing different clusters.

**Figure S3. Cell cycle phases predicted by cycling genes. (A)** The artificial doublets predicted by Doublefinder. **(B-D)** To examine cell cycle variation in our data, we assigned each cell a score, based on its expression of G2/M and S phase markers. t-SNE plots of each cell were shown for MSCs of **(B)** multiple tissues and **(C)** each tissue, respectively. **(D)** t-SNE plots of intra-tissue clusters for each tissue were shown, respectively.

**Figure S4. Heterogeneous characterization of conserved and tissue-specific subpopulations. (A)** t-SNE plot of MSCs from multiple tissues, color coded for each donor. Adipose (A01-A03), Bone marrow (B01-B03), Dermis (D01-D03) and Umbilical cords (U01-U02). **(B)** Circos image showing signature genes from each cluster overlapping. On the outside, each arc represents the identity of each cluster. On the inside, dark orange color represents the genes that appear in multiple clusters and light orange color represents genes that are unique to that cluster. Purple lines link the same gene that are shared by multiple clusters. **(C-D)** Expression patterns of highly DEGs involved in secreted factors (**C**) and core matrisome (**D**) pathways. Each dot represents one gene, of which the color saturation represents the average expression level (scaled by Z-score), connected with Figure 3.

**Figure S5. Effects of MPP1 and MFAP2 knockdown on the expression of immunosuppressive genes**. Quantitative real-time PCR analysis showing relative transcript expression of IDO, PDL1, and CTLA4 in AMSC **(A)**, BMSC **(B)**, DMSC **(C)** and UMSC **(D)** for MPP1 knockdown; AMSC **(E)**, BMSC **(F)**, DMSC **(G)** and UMSC **(H)** for MFAP2 knockdown. The expression of CTLA4 was too low in UMSCs so that could not be detected within 40 cycles by quantitative real-time PCR. (n = 3, mean + SEM, *p < 0.05, **p < 0.01, ***p < 0.001 by two-way ANOVA test). NC representing knockdown scramble control, siRNA1 or siRNA2 representing two different targeting sites, respectively. Color representing the indicated genes.

**Figure S6. Knockdown of MFAP2 altering the expression of osteogenic and adipogenic genes in four types of MSCs.** Quantitative real-time PCR analysis showing relative transcript expression of the osteogenesis and adipogenesis maker genes in AMSC **(A)**, BMSC **(B)**, DMSC **(C)** and UMSC **(D)**. NC representing knockdown scramble control, siRNA1 or siRNA2 representing two different targeting sites, respectively. Color representing the indicated genes. (n = 3, mean + SEM, *p < 0.05, **p < 0.01, ***p < 0.001 by two-way ANOVA test).

**Figure S7. Knockdown of MMP1 altering the expression of osteogenic and adipogenic genes in four types of MSCs.** Quantitative real-time PCR analysis showing relative transcript expression of the osteogenesis and adipogenesis maker genes in AMSC **(A)**, BMSC **(B)**, DMSC **(C)** and UMSC **(D)**. NC representing knockdown scramble control, siRNA1 or siRNA2 representing two different targeting sites, respectively. Color representing the indicated genes. (n = 3, mean + SEM, *p < 0.05, **p < 0.01, ***p < 0.001 by two-way ANOVA test).

**Figure S8. Donor variations and passage effect on the gene expression. (A-B)** Knockdown efficiency of siRNAs for MPP1(**A**) and MFAP2 (**B**) was detected by quantitative real-time PCR. NC representing knockdown scramble control, siRNA1 or siRNA2 representing two different targeting sites, respectively. (**C**) The relative transcript expression of indicated genes related to lineage-differentiation (adipogenesis, osteogenesis) and immunosuppression in different donors, related to **Figure S5-S7**. Three donors in each type of MSCs (n = 3, mean + SEM, *p < 0.05, **p < 0.01, ***p < 0.001 by two-way ANOVA test). Color representing indicated genes. (**D-E**) One open-access scRNAseq dataset for one UMSC sample was integrated to investigate UMSC heterogeneity. tSNE plot depicting all UMSCs colored by sample (**D**) or cluster (**E**). **(F)** Pearson correlation analysis of three passages (P1, P2, and P3) in one BMSC sample.

**Figure S9. The expression of genes via signaling transduction responding to multiple stimulus molecules. (A-B)** Row-normalized heatmap of HVGs within each subpopulation, involving in signaling transduction responding to multiple stimulus molecules, such as PDGFB, BSG and TGFB1(**A**) or SPP1, VEGFA and FGF1(**B**). **(C)** Gene ontology analysis of these genes in tissue-specific subpopulations. The representative terms from the full clusters converted into a network layout. Each term is represented by a circle node, where its size is proportional to the number of input genes fall into that term, and its color represent its cluster identity. The network is visualized with Cytoscape (v3.7.1) with “force-directed” layout and with edge bundled for clarity. (**D**) Gene ontology displaying by tissue-specific subpopulations. Color encoding pie sector represents different tissue-specific subpopulations.

**Figure S10. Representative functional pathways related to immune response.** The full statistically enriched terms were identified, and then accumulative hypergeometric p-values and enrichment factors were calculated and used for filtering. The significant top terms related to immune response were then hierarchically clustered into a tree based on Kappa-statistical similarities.

**Figure S11. Characterization of MHC mediated antigen processing complex in tissue-specific and conserved subpopulations.** Average subpopulation expression of MHC class I **(A)** and class II related genes **(B)**. **(C)** Protein-protein interaction networks of MHC class I and class II related proteins. Protein interaction was combinedly predicted by BioGrid, InWeb_IM and OmniPath database. Colors encoding to distinguish MHC class I and class II.

**Figure S12. Donor variations on the expression of aging and inflammatory associated genes.** Using GSEA database to screen aging and anti-aging related genes. Heatmap showing the relative expression of aging regulatory genes **(A)**, down- or up-regulated ECM genes during aging **(B)**, anti-inflammatory genes **(C),** and inflammatory genes **(D)**. The color bar representing the relative gene expression.

**Figure S13. Gene Expression Differences of MHC-mediated antigen processing complex among different donors.** Average expression of MHC class I **(A)** and class II related genes **(B)** in different donors. **(C-D)** Correlation plots between each two-individual donor on the gene expression in MHC class I **(C)** and class II **(D)** complex pathways, respectively. Color legend (blue to red) encoding the value from 0 to 1.

**Figure S14.** **Inter-donor MSC heterogeneity of developmental trajectories and potential differentiation ability. (A)** Violin plot showing trilineage differentiation genes of MSCs for individual cells from different donors. The black dots representing the values for individual cells. **(B)** Single cell trajectory analysis of MSCs from each tissue by Monocle2. Arrows were drawn from stemness to the differentiating cell lineages over pseudotime. Color encoding individual donor for each tissue.

**Figure S15. Molecular characterization of AMSC subpopulations. (A)** tSNE plot of AMSCs, color-coded for subpopulations. **(B)** Heatmap showing the expression of the top 10 marker genes in subpopulations. **(C)** tSNE plots showing the expression of HMMR, COL15A1, HLA-DRA and CDCP1. **(D)** Protein-protein interaction analysis on signature genes with adjusted P value <0.01, and log (fold change)> 0.25 from each subpopulation. Color encoded for subpopulations.

**Figure S16. Molecular characterization of BMSC subpopulations. (A)** tSNE plot of BMSCs, color-coded for subpopulations. **(B)** Heatmap showing the expression of the top 10 marker genes in subpopulations. **(C)** tSNE plots showing the expression of ITGB8, HLA-DMB, TMEM176A and TMEM176B. **(D)** Protein-protein interaction analysis on signature genes with adjusted P value <0.01, and log (fold change)> 0.25 from each subpopulation. Color encoding for subpopulations.

**Figure S17. Molecular characterization of DMSC subpopulations. (A)** tSNE plot of DMSCs, color-coded for subpopulations. **(B)** Heatmap showing the expression of the top 10 marker genes in subpopulations. **(C)** Protein-protein interaction analysis on signature genes with adjusted P value <0.01, and log (fold change)> 0.25 from each subpopulation. Color encoding for subpopulations. **(D)** tSNE plots showing the expression of TMEM35, NGFR, ITGA7, HMMR, TMEM176B, TINAGL1, MGP and COL11A1.

**Figure S18. Molecular characterization of UMSC subpopulations. (A)** tSNE plot of UMSCs, color-coded for subpopulations. **(B)** Heatmap showing the expression of the top 10 marker genes in subpopulations. **(C)** tSNE plots showing the expression of DPT, COL15A1, CLDN11 and TIMP3. **(D)** Protein-protein interaction analysis on signature genes with adjusted P value <0.01, and log (fold change)> 0.25 from each subpopulation. Color encoding for subpopulations.

**Figure S19. Distinct functional clusters showing inter-tissue heterogeneity using PPI network analysis.** PPI network of signature genes in each tissue-specific subset was investigated using BioGrid, InWeb_IM, and OmniPath combined database. The top 3 enriched network for each tissue was shown, respectively.

**Figure S20. Intra-tissue MSC heterogeneity in developmental trajectories.** (**A-D**) RNA velocities were visualized on the t-SNE projection of MSCs from multiple donors using Gaussian smoothing on a regular grid for each tissue. **(A)** Adipose, **(B)** Bone marrow, **(C)** Dermis, **(D)** Umbilical cords, respectively. (**E**) Single cell trajectory analysis by Monocle2 on individual subpopulations for each tissue (adipose, bone marrow, dermis, umbilical cords). Color encoding each subpopulation. Arrows were drawn from stemness to the differentiating cell lineages.

**Figure S21. Hematopoietic associated factors clustering by pseudo-temporal expression patterns in each tissue. (A-D)** Heatmap depicting the expression of hematopoietic associated factors along temporal distribution in pseudotime for adipose **(A)**, bone marrow **(B)**, dermis **(C)**, and umbilical cord **(D)**, respectively. Genes were hierarchically clustered based on pseudotime-dependent expression. The genes were scaled as the percentage of maximum expression. **(E-F)** Expression levels of CCL2 **(E)** and CXCL12 **(F)** in total cells from each tissue with respect to their pseudo-time coordinates. Black lines depict the LOESS regression fit of the normalized expression values.

**SUPPLEMENTARY TABLE INFORMATION**

**Supplementary Table 1.** The information of samples used for single cell RNAseq.

**Supplementary Table 2**. Top-10-ranking differential expressed genes in each individual clusters.

**Supplementary Table 3.** Gene Ontology (GO) analysis of the predominantly significant genes from each individual clusters.

**Supplementary Table 4.** Average expression of total DEGs in each individual cluster, identified by each cluster vs the other clusters.

**Supplementary Table 5.** Protein-protein interactions analysis on tissue-specific subpopulations.
